# Leveraging a KRAS-based signature to predict the prognosis and drug sensitivity of colon cancer and identifying SPINK4 as a new biomarker

**DOI:** 10.1038/s41598-023-48768-0

**Published:** 2023-12-14

**Authors:** Jian-Ting Huo, Abudumaimaitijiang Tuersun, Su-Yue Yu, Yu-Chen Zhang, Wen-Qing Feng, Zhuo-Qing Xu, Jing-Kun Zhao, Ya-Ping Zong, Ai-Guo Lu

**Affiliations:** Department of General Surgery, Shanghai Minimally Invasive Surgery Center, Ruijin Hospital, Shanghai Jiao Tong University School of Medicine, Shanghai, 200020 People’s Republic of China

**Keywords:** Gastrointestinal cancer, Computational biology and bioinformatics, Genetics

## Abstract

KRAS is one of the leading mutations reported in colon cancer. However, there are few studies on the application of KRAS related signature in predicting prognosis and drug sensitivity of colon cancer patient. We identified KRAS related differentially expressed genes (DEGs) using The Cancer Genome Atlas (TCGA) database. A signature closely related to overall survival was recognized with Kaplan–Meier survival analysis and univariate cox regression analysis. Then we validated this signature with overall expression score (OE score) algorithm using both scRNA-seq and bulk RNA-seq data. Based on this signature, we performed LASSO cox regression to establish a prognostic model, and corresponding scores were calculated. Differences in genomic alteration, immune microenvironment, drug sensitivity between high- and low-KRD score groups were investigated. A KRAS related signature composed of 80 DEGs in colon cancer were recognized, among which 19 genes were selected to construct a prognostic model. This KRAS related signature was significantly correlated with worse prognosis. Furthermore, patients who scored lower in the prognostic model presented a higher likelihood of responding to chemotherapy, targeted therapy and immunotherapy. Furthermore, among the 19 selected genes in the model, SPINK4 was identified as an independent prognostic biomarker. Further validation in vitro indicated the knockdown of SPINK4 promoted the proliferation and migration of SW48 cells. In conclusion, a novel KRAS related signature was identified and validated based on clinical and genomic information from TCGA and GEO databases. The signature was proved to regulate genomic alteration, immune microenvironment and drug sensitivity in colon cancer, and thus might serve as a predictor for individual prognosis and treatment.

## Introduction

Colorectal cancer is the second leading cause of cancer-related mortality worldwide, with about 1.9 million new cases and 935,000 deaths in 2020^1^. Surgery is still the primary treatment for colon cancer patients without distant metastasis. However, for metastatic colorectal cancer (mCRC), featured by a poor prognosis with a median overall survival of only 25–30 months^2^, the therapy remains a big challenge. The development of targeted therapy has brought new breakthroughs to the treatment of mCRC, and researches about combination therapy with targeted agent (anti-EGFR, epidermal growth factor receptor; anti-VEGF, vascular endothelial growth factor) constantly emerge^3–5^. However, targeted therapy in CRC is restricted by the heterogeneity between individuals. Meanwhile, immune checkpoint blockade (ICB) therapy has been widely used in patients with advanced malignant cancer including non-small cell lung cancer, melanoma, renal cell carcinoma and other mismatch repair-deficient tumors with promising/desirable therapeutic effect^6^. ICB therapy is also recommended in the treatment of mCRC patients with MisMatch repair-deficient/MicroSatellite instability-high (dMMR/MSI-H)^7^. Unfortunately, the proportion of dMMR/MSI-H in stage IV CRC is only 2.1–4%^8–11^ which limit the further application of immunotherapy.

The KRAS gene is one of the most frequent mutations in CRC^12^. It is a member of the rat sarcoma viral oncogene family (RAS) with other two isoforms: HRAS and NRAS. About 30–50% CRC cases had KRAS mutation, among which G12D and G12V are the most two common mutation subtypes^12–15^. The gene encodes the KRAS protein, which works as a molecular switch controlling multiple downstream signaling cascades by changing between activated and inactivated state. Mutated KRAS, however, alters the protein to impede the deactivation of KRAS, resulting in the constitutive activation of KRAS^16,17^. The persistent activation of KRAS downstream effectors lead to the occurrence of malignant transformation^18^.

KRAS mutations are of pivotal clinical importance because they are widespread in cancer and play a decisive part in inducing tumorigenesis^18^. These mutations affect treatment strategies and represent a significant therapeutic target for oncological advancements. Therefore, KRAS mutations encompass a broad scope of clinical applications, one of the critical implications of KRAS mutations is their role in resistance to targeted therapies. KRAS mutation can lead to the resistance to anti-EGFR therapy such as cetuximab and panitumumab due to the constitutively activation of KRAS protein^19,20^. Besides, mutations at different codons also result in difference in treatment response, as a recently emerging agent targeting KRAS-G12C mutation which only accounts for 4% of all patients could not work for other KRAS mutation subtypes^21^. As for other anti-tumor therapy, it was also reported that patients with KRAS G13D usually presented an inferior response to chemotherapy^22^. Besides, KRAS mutations contribute to forming immunosuppressive tumor microenvironment (TME) and modifying immune cells, resulting in tumor immune escape^23,24^, and thus potentially reduce the effectiveness of immunotherapy.

Testing for KRAS mutations has become a routine practice in the diagnosis and treatment of CRC. Nevertheless, the evidence regarding the prognostic value of KRAS mutations remains conflicting. KRAS mutations were associated with poor prognosis in metastatic CRC patients^14^. In localized CRC patient, however, the results from different studies are inconsistent. A clinical trial reported that KRAS mutation did not affect overall survival in stage II/III colon cancer^25^, while others documented a negative impact of KRAS mutation on overall survival^26–28^. It was probably different KRAS subtypes that contribute to different outcomes^29^, but the specific mechanism has not been fully elucidated.

The mutation status of KRAS alone is an insufficient indicator of anti-tumor drug resistance as well as overall prognosis, and its specific role remains unclear. Heterogenous evidence from current studies indicated the possibility of a set of KRAS-related genetic signature, rather than KRAS mutation alone, which may better recognize and characterize a group of CRC patients with similar intrinsic patterns. However, limited research has been conducted on KRAS related signature as well as its efficacy in determining patients’ prognosis and informing the clinical medication.

In this study, clinical and genomic information from TCGA and GEO databases were analyzed to identify a KRAS related signature. Based on this, a prognostic model related to KRAS mutation was established to quantify individualized KRAS-related patterns in colon cancer patients, and to predict prognosis and personal response to drugs and therapies commonly used in clinical practice.

## Methods

### Data preparation and preprocessing

Public databases including The Cancer Genome Atlas (TCGA, https://cancergenome.nih.gov/) and NCBI Gene Expression Omnibus (GEO, https://www.ncbi.nlm.nih.gov/geo/) were used to collect the gene expression and clinical characteristics of colon cancer samples. R package “TCGAbiolinks” was used to obtain data from TCGA. Raw count and transcripts per kilobase million (TPM) values were used for different analyses accordingly. Human endogenous retroviruses (HERVs) infection related data were downloaded from the supplementation of a recent study by Elbasir A et al.^30^.

### Identification of differentially expressed genes (DEGs) between KRAS mutated and wildtype COAD tissues

Briefly, the differentially expressed genes (DEGs) related to KRAS mutation in COAD were identified in two steps. Firstly, 481 patients from the TCGA database were separated into two groups according to their KRAS mutation status. Then DEGs between two groups were recognized using R package “limma” with an adjusted *p* value < 0.01 and a |logFC|> 1. Secondly, Kaplan–Meier survival analysis and univariate cox regression analysis were used to screen survival related DEGs, adjusted *p* value < 0.05 was set as the criterion for both analyses.

### Overall expression (OE) of KRAS related DEGs

To quantitative the level of KRAS related DEGs in each cell or bulk sample, we used an algorithm called gene set overall expression (OE) reported by Jerby-Arnon et al.^31,32^. This algorithm using given gene signatures and expression matrix to determine the expression level of gene modules. And this algorithm takes into account differences in the signal-to-noise ratio across genes and cells.

Firstly, for a given expression matrix $\mathrm{C}$, bulk RNA-seq data were normalized and scRNA-seq data were transformed into TPM. The average expression of gene ${E}_{i}$ is calculated as:

$$E_{i} = \left\{ {\begin{array}{*{20}c} {\frac{{\mathop \sum \nolimits_{j} C_{i,j} }}{N},} & {j = 1, \ldots ,N; \,bulk \,RNAseq} \\ {log_{2}}{\left ( {\frac{{\mathop \sum \nolimits_{j} 10 \times \left( {2^{{C_{i,j} }} - 1} \right)}}{N} + 1} \right),} & {j = 1, \ldots ,N;scRNAseq} \\ \end{array} } \right.$$

Secondly, all genes were binned into 50 expression bins based on their ${E}_{i}$ across all samples. Then, the number of occurrences of concerned gene set $\mathrm{K}$ was counted as their frequency within each bin, respectively. Random signature $\mathrm{K{\prime}}$ which shared the same frequency in each bin with gene set $\mathrm{S}$ were randomly generated and 1000 repetitions were conducted.

To avoid the domination effect of genes with excessive expression level, a centered gene expression matrix $\mathrm{Z}$ was defined as:

$${Z}_{i,j}={C}_{i,j}-\frac{{\sum }_{j}{C}_{i,j}}{N},j=1,\dots ,N$$

For a given gene set, the score of each patient or cell was defined as the mean of centered expression value of each gene. Then, the scores of $\mathrm{K}$ (${S}_{K}$) and ${\text{K}}^{\prime }$ ($S_{{K^{\prime } }}$) were calculated correspondingly. Finally, the OE score of gene set $\mathrm{K}$ was defined as:

$$\mathrm{OE}={S}_{K}-\frac{{\sum }_{n}{S}_{{K{\prime}}_{n}}}{1000},n=1,\dots ,1000$$

The 80 KRAS related DEGs as screened previously were divided into two groups, of which one is upregulated (${OE}_{up}$) in KRAS mutated patient and the other is downregulated (${OE}_{down}$). We defined the OE score of KRAS related DEGs as:

$${OE}_{KRAS}={OE}_{up}-{OE}_{down}$$

### Unsupervised consensus clustering

The R package “ConsensusClusterPlus” was utilized for the clustering based on KRAS related DEGs. K = 10 was set as the maximum number of clusters, and 1000 repetitions were conducted to ensure the stability and repeatability.

### Construction and validation of prognostic model

Lasso Cox regression analysis was performed on survival related DEGs. Nineteen genes were finally screened and involved to construct the prognostic model, and the KRAS related DEGs score (KRD score) was calculated as the formula:

$$\mathrm{score}=\sum \left({Exp}_{i}\times {Coef}_{i}\right)$$

${Coef}_{i}$ represented the coefficient of genes, and ${Exp}_{i}$ denoted the gene expression. Patients were divided into high and low score group with the median score as a cut point.

### Enrichment analysis and functional annotation

Single sample gene set enrichment analysis (ssGSEA) was performed to investigate the heterogeneity of different groups using R package “GSVA”. Gene sets “h.all.v7.4.symbols.gmt” was obtained from Molecular signatures database (MsigDB, http://www.gsea-msigdb.org/) database for enrichment analysis. For single cell data, R package “irGSEA” was utilized to estimate the pathway activity of each cell.

### Mutation landscape and drug sensitivity analysis

The mutation annotation format was conducted using R package “maftools” and tumor mutation burden (TMB) of each patient was extracted from The Cancer Immunome Atlas (TCIA, https://tcia.at/). R package “pRRophetic”was applied to predict the drug sensitivity of each patient. IC50s to drugs and gene expression data of different cell lines were obtained from Broad Institute Cancer Cell Line Encyclopedia (CCLE, https://portals.broadinstitute.org/ccle/about) for further validation.

### Tumor microenvironment analysis and immunotherapy response prediction

ESTIMATE algorithm was used to calculate the immune score, ESTIMATE score and stromal score of each patient. Immune cell infiltration analysis was conducted by CIBERSORTx website (https://cibersortx.stanford.edu/) with default parameters. Data of T cell dysfunction, T cell exclusion and TIDE scores were calculated from TIDE website (http://tide.dfci.harvard.edu/). Immunophenoscore (IPS) data of each patient in TCGA-COAD program was downloaded from TCIA database. To further validate the models’ efficacy to predict the immunotherapy response, two independent immunotherapeutic cohorts were included in our study: melanoma treated with anti-CTLA4 and anti-PD1 antibody (GSE91061); advanced urothelial cancer treated with atezolizumab (IMvigor210 cohort). The response of TCGA cohort were predicted by TIDE website.

### Single-cell RNA-seq analysis

Dataset GSE166555 was downloaded from the GEO database. The annotation profile was extracted from TISCH2 website (http://tisch.comp-genomics.org/). R package “Seurat”, which is a widely utilized single cell transcriptome analysis tool, was used to analyze the single-cell dataset. The “cellchat” and “nichenet” R packages were used to infer the interactions between different cell types with default parameters^33^.

### Cell line culture

Cell lines SW48 were obtained from the American Type Culture Collection (ATCC, Manassas, VA) and stored at the Shanghai Institute of Digestive Surgery. Cells were cultured in RPMI-1640 (Gibco, Grand Island, NY, USA) supplemented with 10% fetal bovine serum (FBS), 1% penicillin/streptomycin (Gibco) at 37 °C with 5% CO_2_.

### SPINK4 knockdown

SW48 cells were plated in 6-well plates at a density of 50,000 cells per well. After 24 h culture, the medium was replaced with fresh culture medium. Cells were transfected with siRNA targeting SPINK4 or nontargeted control siRNA (Genomeditech, CHINA) at 37 °C for 2 h, then the culture medium was refreshed for another 24 h.

### RNA extraction and quantitative real-time PCR (qRT-PCR)

Total RNA was extracted using TRIzol (Invitrogen, USA). And a spectrophotometer (BioTek, Vermont, USA) was used to evaluate the quality and concentration of RNA. Then total RNA was reversed to cDNA by HiScript III RT SuperMix (Vazyme, China) and SYBR Green (Vazyme, China) was used to perform qPCR accordingly. The relative expression of mRNAs was calculated by the 2-ΔΔCT method.

### Cell viability analysis

SW48 cells were seeded in 96-well plates with a density of 3000/well. Cell counting kit8 (CCK8, Dojindo, Kumamoto, Japan) was utilized to conduct cell proliferation assay. According to the manufacture’s protocol, cck8 was added into the culture medium with the final concentration at 10% for 2 h, then OD450 was measured by spectrophotometry (BioTek, Vermont, USA).

### Wound healing assay and transwell assay

Cells were seeded in 6-well plates to prepare a confluent cell monolayer, then scratches were made with a pipette tip. Cells were subsequently photographed per 12 h. For transwell assay, cells were seeded in upper chamber in RPMI 1640 without FBS at a density of 60,000/well. The lower chamber was filled with 800μL of RPMI 1640 medium with 20% FBS contained. After 48 h incubation, cells were fixed and stained with crystal violet. ImageJ software was utilized to analyze the migrated areas and count the migrated cells.

### Statistical analysis

The statistical analysis was performed using R 4.1.2 software. For quantitative data, Student’s test and Wilcoxon test were used respectively in normally distributed or non-normally distributed variables. For comparisons among three groups or more, the statistical significance of parametric methods was estimated by one-way analysis of variance, and non-parametric methods were analyzed using Kruskal–Wallis test. For survival analysis, Kaplan–Meier analysis was performed. Statistical significance level was a two-tailed alpha of 0.05. When analyzing genomic data, Benjamini–Hochberg method was performed for multiple hypotheses testing.

### Ethical approval and consent to participate

This study was approved by the Institutional Review Board of Ruijin Hospital Ethics Committee (Shanghai Jiao Tong University School of Medicine).

## Results

### Identification of differential expression genes between KRAS mt and KRAS wt colon cancer tissues and the landscape of their genetic variation

**Figure 1:**
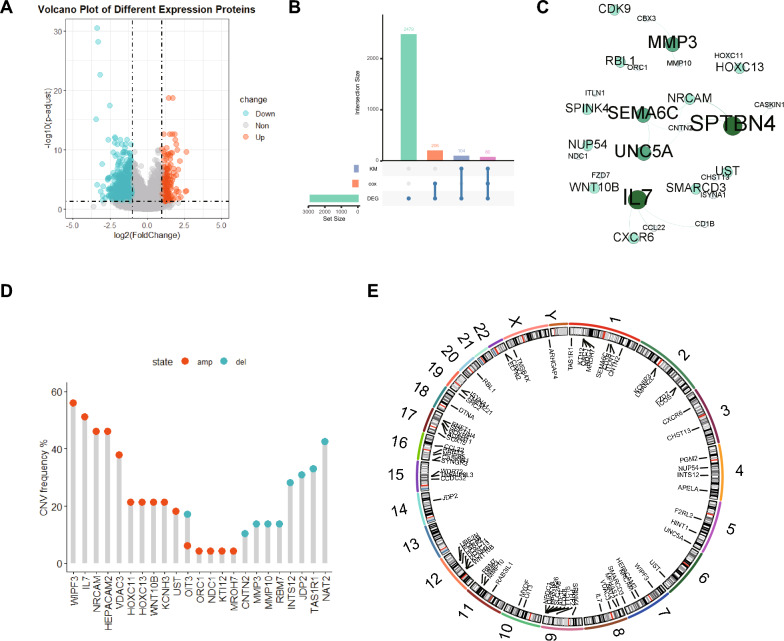
Identification of differential expression genes between KRASmt and KRASwt colon caner tissues and the landscape of their genetic variation. (**A**) Volcano plot shows differentially expressed genes from KRAS mutation and KRAS wildtype patients. Significantly regulated genes in KRAS mutation group are shown in red (upregulated) or cyan (downregulated). (**B**) Upset plot of totally 2869 differentially expressed genes identified between KRASmt and KRASwt colon cancer tissues. (**C**) PPI network of KRGs based on STRING database. (**D**) The lollipop plot of CNV frequency of 80 KRGs (**E**) The location of CNV alteration of 80 KRGs on chromosomes.

We included 190 KRAS mutation (KRAS mt) colon cancer and 291 KRAS wildtype (KRAS wt) colon cancer tissue samples obtained from the TCGA-COAD cohort. Differential expression analysis was applied with | log2FC |> 1, and statistical significance level was *p* < 0.01. In total, 2869 differentially expressed genes were identified (Fig. 1A). We performed Kaplan–Meier analysis and univariate cox regression analysis to recognize prognosis related genes, and finally 80 KRAS related DEGs (KRDs) were retained (Fig. 1B, S1D, Table S1). The expression of these genes in two groups were summarized in Figure S1A. The alteration of 80 KRDs was summarized in Figure S1B. As shown in Fig. 1C, the protein–protein interaction (PPI) network of these genes were constructed. We then analyzed the frequency of CNV alteration of these genes, of which 15 suffering CNV amplification while 9 genes suffering deletion (Fig. 1D). Then, the location of these genes was visualized (Fig. 1E).

### Analysis of KRAS related DEGs at the level of single cell

**Figure 2:**
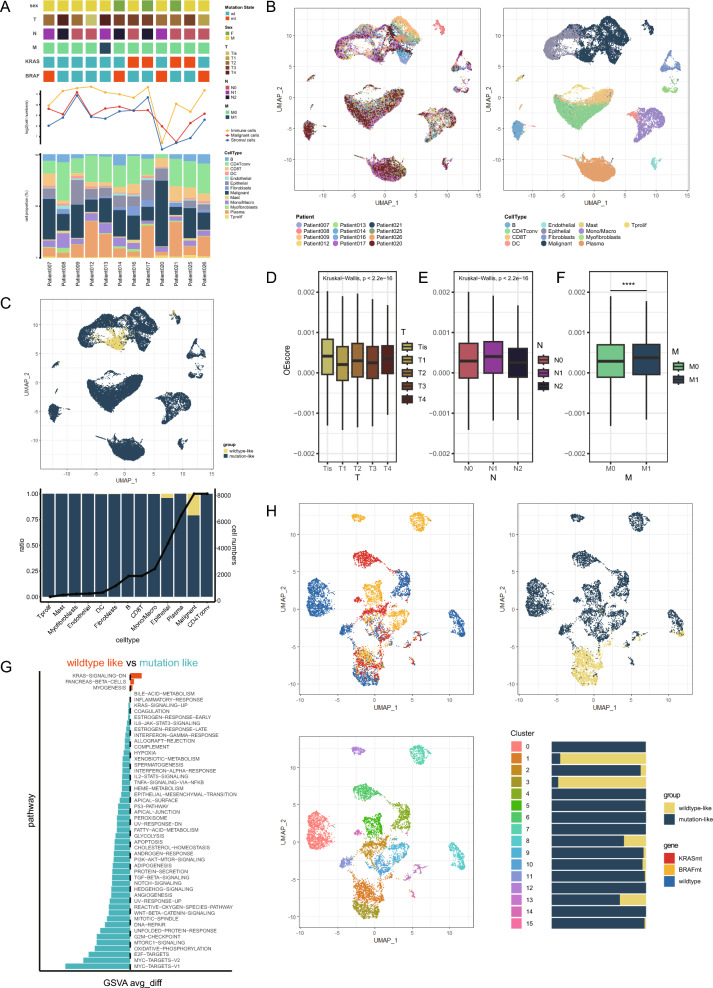
Analysis of KRAS related DEGs at the level of single cell. (**A**) Clinical and molecular feature of 12 colon cancer patients from GSE166555 (upper). Cell numbers of malignant cells, immune cells and stromal cells (middle). The proportion of different cell types from each patient (lower). (**B**) Uniform manifold approximation and projection (UMAP) of total 36,822 cells is colored in terms of individual patients (left) or cell types (right). (**C**) UMAP of total cells (upper). The proportion of wildtype-like cells and mutation-like cells in each cell types, line graph represent the number of each cell types (lower). (**D**–**F**) The KRAS OE score in samples of different pathological stage including T stage (D), N stage (**E**), M stage (**F**). Statistical differences between groups were calculated by Wilcox-rank test. ****p < 0.0001. (**G**) Barplot of significantly activated pathways in mutation-like cells and wildtype-like cells. (**H**) UMAP of malignant cells. The distribution of different sample origins (upper, left). The distribution of wildtype-like cells and mutation-like cells (upper, right). The 16 clusters of malignant cells identified (lower, left). The proportion of wildtype-like and mutation-like cells of each cluster (lower, right).

To validate if KRDs can represent the KRAS mutation status in colon cancer, we obtained a well-annotated single-cell RNA sequence dataset (GSE166555) from GEO database. The annotation profile was downloaded from TISCH2 website. 12 Tumor samples composed of KRAS mutated group (n = 4), BRAF mutated group(n = 4) and wildtype group (n = 4) were harvested from 12 patients (Fig. 2A). After quality control filtering, 36,822 cells distributed across the twelve samples were kept for further analysis. Uniform manifold approximation and projection (UMAP)‐based clusters were displayed in Fig. 2B. UMAP exhibited that the malignant cells practically formed distinct clusters corresponding to their sample source, whereas nonmalignant cells displayed similar patterns across all twelve patients.

Next, 80 KRDs were selected to calculate the score of KRAS signature. A scoring algorithm called overall expression score (OE score) were used to quantify KRD score in all cells. The algorithm employs prescribed gene signatures as well as expression matrix, facilitating the precise estimation of gene module expression levels. Calculated overall expression scores approximate a binary normal distribution (Figure S2A). The cells were then divided into two groups with a cut-off of − 0.0025. Cells exhibiting a relatively diminished score were classified as "wildtype-like" cells, while those surpassing this threshold were categorized as "mutation-like" cells. As shown in Fig. 2C, mutation-like cells only distributed in malignant cells and epithelial cells. Furthermore, malignant cells were isolated and divided into 16 clusters using UMAP algorithm (Fig. 2H). Wildtype-like cells mainly distributed in cluster1 and cluster3, and mutation-like cells originated from KRAS mutated, BRAF mutated and wildtype patients evenly (Figure S2B). Differentially expressed genes between mutation-like and wildtype-like malignant cells were identified (Figure S2C). The KRAS OE scores showed heterogeneity among different patients and cell types (Figure S2F, S2G). We compared the OE score in different pathology stages, and the results indicated that OE score differed across T and N stages without a clear trend. However, cells obtained from M1 stage patients presented a significantly higher KRAS OE score which suggested KRAS signature was related to distant metastasis of colon cancer (Figs. 2D, E, F, S2E). Then gene set variation analysis (GSVA) was performed among all malignant cells with HALLMARK gene sets, and the results revealed most pathways including KRAS-SIGNALING-UP pathway was enriched in mutation like malignant cells group, while only three pathways were significantly upregulated in wildtype like malignant cells group: PANCREAS-BETA-CELLS/KRAS-SIGNALING-DN/MYOGENESIS (Fig. 2G). The results indicated that mutation like malignant cells got a globally higher activity, and to some extent, the KRAS OE score can reflects the changes at the transcriptome level caused by KRAS mutation.

### Cell–cell interactions within TME were remodeled by KRAS OE score

**Figure 3:**
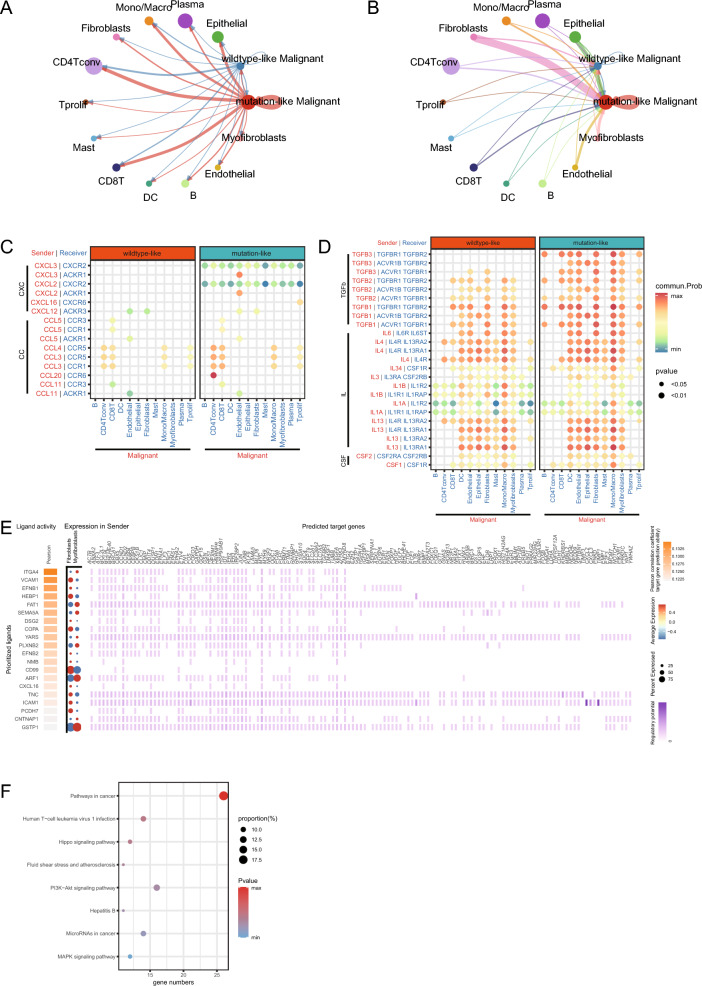
Cell–cell interactions within TME were remodeled by KRAS OE score. (**A**) The outgoing signals of malignant cells, the thickness of lines denotes the interaction weight. (**B**) The incoming signals of malignant cells, the thickness of lines denotes the interaction weight. (**C**–**D**) Interactions between malignant cells and other cells through ligand–receptor (LRs), which includes chemokines (**C**) and other cytokines (**D**). (**E**) Expression of top 20 active ligands from fibroblasts and the predicted target genes in malignant cells. (**F**) KEGG enrichment analysis of predicted target genes in malignant cells.

Furthermore, to understand the cell–cell interactions between malignant cells and other cells within the TME, we performed cell communication analysis using R package “cellchat”. As shown in figure S3A, S3B, a cell–cell communication network was constructed with weighted incoming/outgoing signals. Mutation-like malignant cells presented higher weight of both incoming and outgoing signals compared with wildtype-like malignant cells (Fig. 3A, B). It was noteworthy that fibroblasts showed the highest weight among all cell types in communication with mutation-like malignant cells. The results indicated that KRAS OE score remodeled the cell communication within TME.

To further understand how KRAS OE score regulate the cell–cell interaction, we analyzed the leading ligand–receptor pairs. The results indicated that KRAS OE score can regulate the ligand–receptor pairs responding for the communication between malignant cells and other cell types to remodel the TME (Table S2). Mutation-like cells secreted higher level chemokines such as CXCL3, CXCL2 to interact with other cell types (Fig. 3C, S3C). KRAS OE score also manipulated the expression of other cytokines, as mutation-like cells presented higher transforming growth factor-β (TGF-β) levels while wildtype-like cells secreted more Interleukin (IL) to communicate with others (Fig. 3D).

We then analyzed the significantly elevated communication from fibroblasts to mutation-like malignant cells with R package “nichenet” (Fig. 3E). The results indicated ligands including ITGA4, VCAM1, EFNB1 etc. were responsible for their communication. The KEGG pathway^34–36^ enrichment analysis of predicted target genes in mutation-like cells showed that fibroblasts could regulate the cancer related genes and activate several signaling pathways in malignant cells (Fig. 3F).

### Validation of the performance of KRAS OE score using bulk RNA-seq datasets

**Figure 4:**
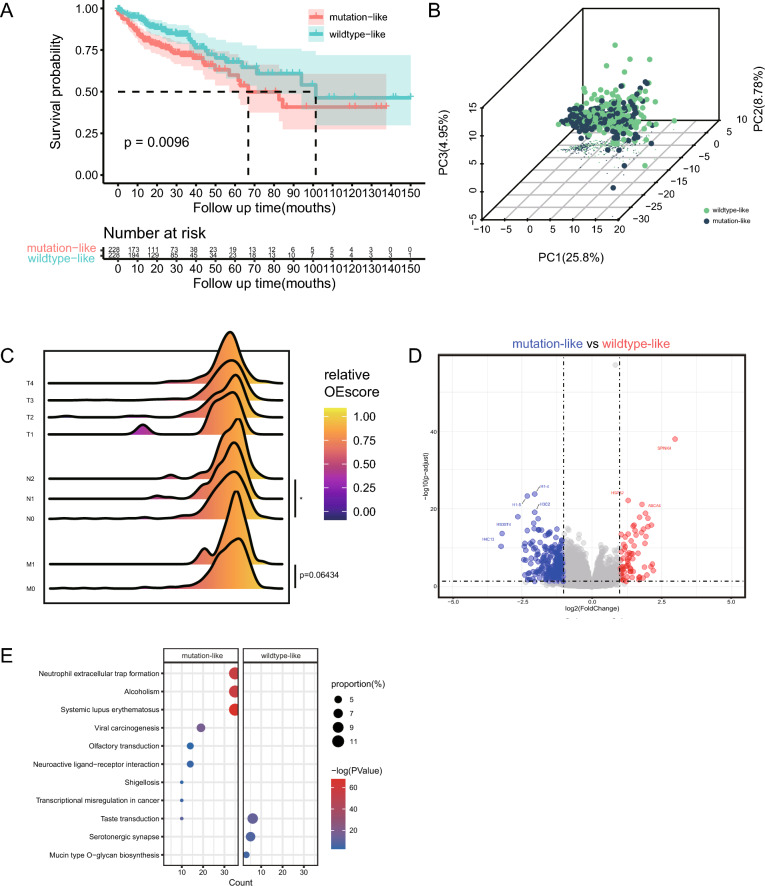
Validation of the performance of KRAS OE score using bulk RNA-seq datasets. (**A**) Kaplan–Meier survival curves for the mutation-like and wildtype-like groups. (**B**) The principal component analysis based on transcriptome profiles of two groups reveals different patterns. (**C**) The KRAS OE score in samples of different pathological stage including T stage, N stage, M stage. Statistical differences between groups were calculated by Wilcox-rank test. ****p < 0.0001. (**D**) The volcano plot shows the differentially expressed genes between mutation-like and wildtype-like groups. (**E**) KEGG enrichment analysis of DEGs in both groups.

We next calculated the KRAS OE score using bulk RNA-seq data from TCGA-COAD project. The distribution of bulk RNA-seq based KRAS OE score was close to normal distribution, and we set the median KRAS OE score as the cut point to divide all patients into two groups (mutation-like and wildtype-like). We performed survival analysis and found that wildtype-like patients showed better survival rate (Fig. 4A). And Principal Component Analysis was conducted for validation (Fig. 4B). We compared the KRAS OE score in patients with different pathological stages, reflecting that patients with distant metastasis had higher OE score (Fig. 4C), which was consistent with our single cell analysis above. Then the differentially expressed genes were identified from two group patients (Fig. 4D), SPINK4 presented the highest fold change. Then the DEGs enrichment analysis was performed (Fig. 4E), we found that DEGs upregulated in mutation-like patients are enriched in neutrophil extracellular trap formation pathway, which has been proved to promote the proliferation, invasion, and metastasis of tumors.

### KRAS related DEGs based molecular subtypes

**Figure 5:**
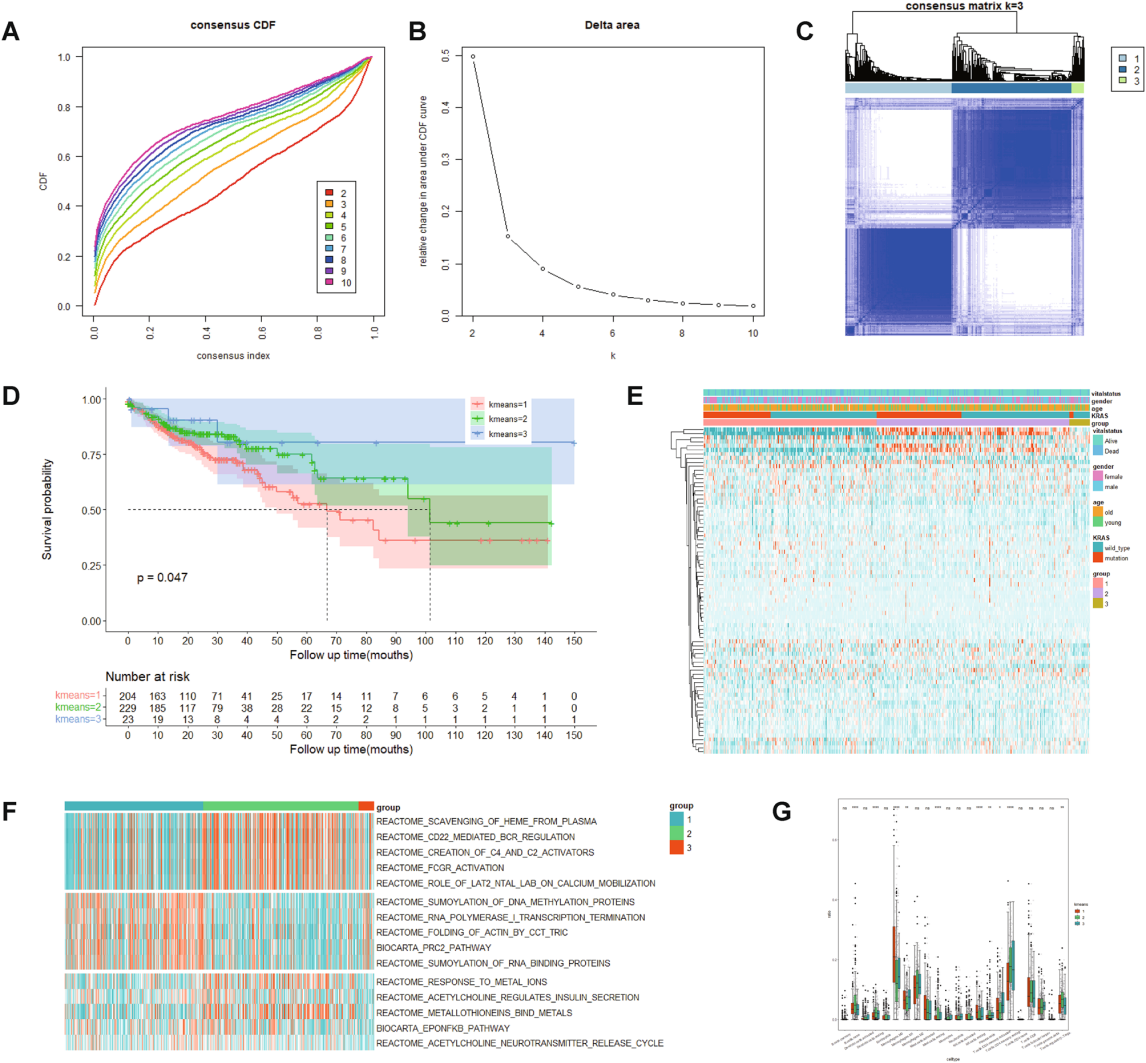
KRAS related DEGs based molecular subtypes. (**A**, **B**) TCGA-COAD samples were analyzed by consistent cluster analysis based on 80 KRDs. (**C**) The consistency matrix heatmap with the k number is 3. (**D**) Kaplan–Meier survival curves of patients with different expression patterns. (**E**) Heatmap of 80 KRDs. (**F**) Differentially enriched pathways identified by GSVA analysis of three molecular subtypes. (**G**) CIBERSORT calculate the immune cell infiltration in three subgroups.

Samples from TCGA-COAD database were divided into different subtypes by consensus clustering based on 80 KRDs to identify sample groups with similar patterns (Fig. 5E). According to the cumulative distribution function (CDF) curve, k = 3 was determined and 430 cases were divided into three groups based on their different expression patterns (Fig. 5A–C). Survival analysis showed that group1 had the worst prognosis while group3 had the best (Fig. 5D). GSVA results indicated difference regarding functional enrichment in three groups (Fig. 5F). Further analysis showed that the infiltration of different immune cell types differed among expression patterns (Fig. 5G).

### Development and validation of a risk model

**Figure 6:**
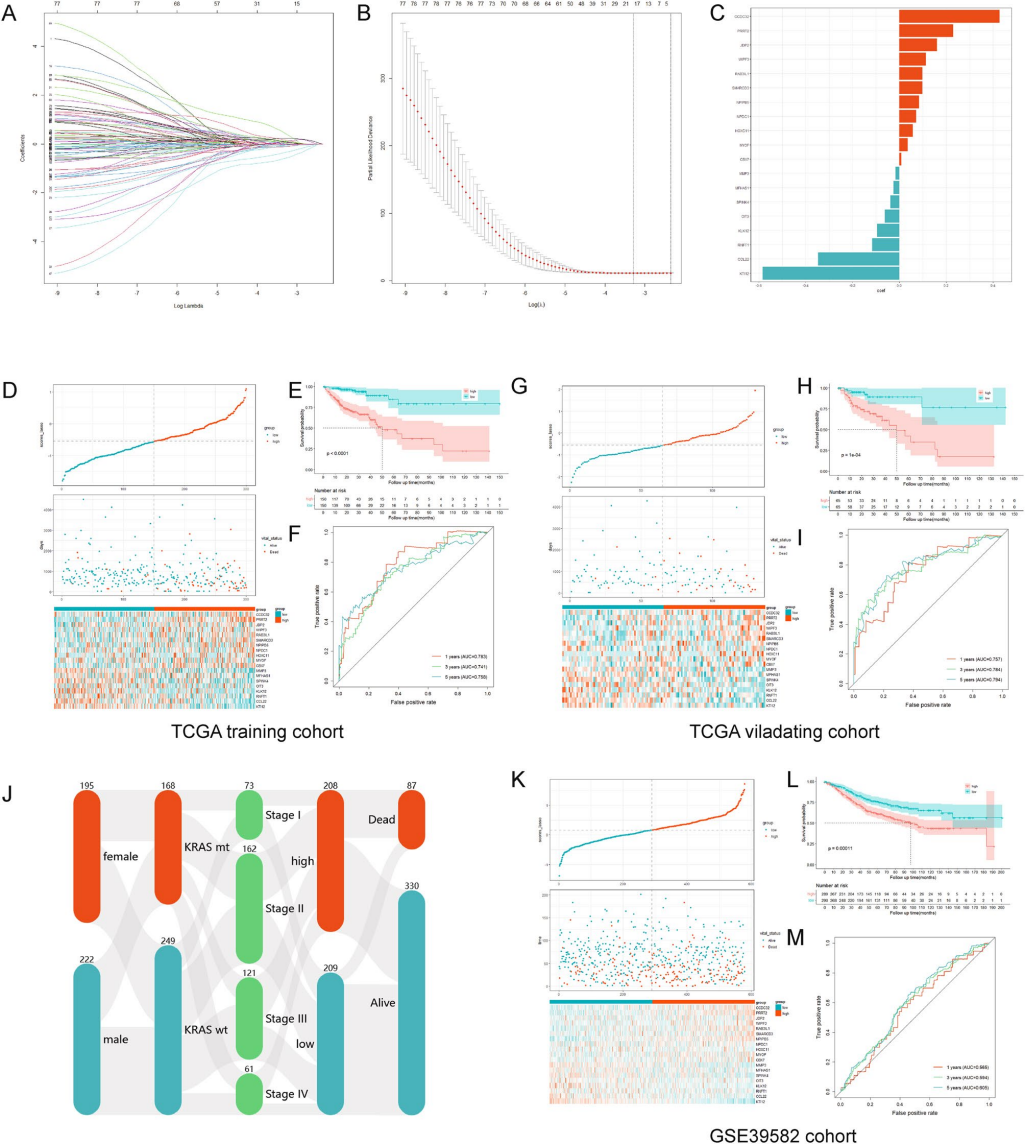
Development and validation of a risk model. (**A**–**B**) LASSO regression analysis of 80 KRDs. (**C**) 19 genes determined based on the optimal value of λ. (**D**) The relationship between survival status/time and KRD score of TCGA training cohort (upper) and mRNA expression heatmap of 80 KRDs (lower). (**E**) Survival analysis for low KRD score and high KRD score groups in TCGA training cohort. (**F**) ROC curves of predicting prognosis in TCGA training cohort. (**G**) The relationship between survival status/time and KRD score of TCGA validating cohort (upper) and mRNA expression heatmap of 80 KRDs (lower). (**H**) Survival analysis for low KRD score and high KRD score groups in TCGA validating cohort. (**I**) ROC curves of predicting prognosis in TCGA validating cohort. (**J**) Sankey plot shows the relationships of sex, KRAS mutation, pathology stage, KRD score group and vital status. (**K**) The relationship between survival status/time and KRD score of GSE39582 cohort (upper) and mRNA expression heatmap of 80 KRDs (lower). (**E**) Survival analysis for low KRD score and high KRD score groups in GSE39582 cohort. (**F**) ROC curves of predicting prognosis in GSE39582 cohort.

We performed lasso regression analysis of the identified 80 survival-related KRDs based on the minimum partial likelihood deviance to develop a KRDs based scoring model (KRD score) for clinical prediction. The model composed of 19 genes, which was determined based on the optimal value of λ (Table S3). The regression coefficient of each gene is displayed in Fig. 6A–C. The KRD score of each patient was calculated by the formula, and all patients were assigned to the high- and low-KRD group divided by the median score. 60% patients from TCGA database were used as training cohort (Fig. 6D–F), and the rest 40% patients served as validation cohort (Fig. 6G–I). KM curve revealed that the survival probability of high-KRD score group patients was significantly lower than that of the low-KRD score group in the training cohort. To evaluate the predictive efficiency of this model in 1-, 3- and 5-year survival rates, time related ROC analysis was conducted. The area under the ROC curve (AUC) is 0.776 at 1 year, 0.755 at 3 years, and 0.775 at 5 years, suggesting that this model was equipped with good prediction efficiency. We also calculated the score of each patient in validation cohort with the same method. Consistent with the training cohort, the results indicated that patients with high-KRD scores got a lower probability of survival than those patients with low-KRD scores. The AUC is 0.783 at 1 year, 0.741 at 3 years, and 0.758 at 5 years. Overall, TCGA cohort presented that patients with a high-KRD score exhibited a poorer prognosis (HR = 2.45, 95% CI 1.66–3.61, *p* < 0.0001). The distribution of patients in TCGA-COAD cohort in high- or low-KRD score group was illustrated in Fig. 6J, with clinical features including sex, KRAS mutation state, pathological stage, outcome information. In addition, GSE39582 was used for further validation (Fig. 6K–M), and similar results were observed as the low-KRD score group presented a better overall survival than high-KRD score group (HR = 1.75, 95% CI 1.32–2.32, *p* = 0.00011). The AUC is 0.565 at 1 year, 0.594 at 3 years, and 0.605 at 5 years.

### The landscape of genomic alterations between different risk groups

**Figure 7:**
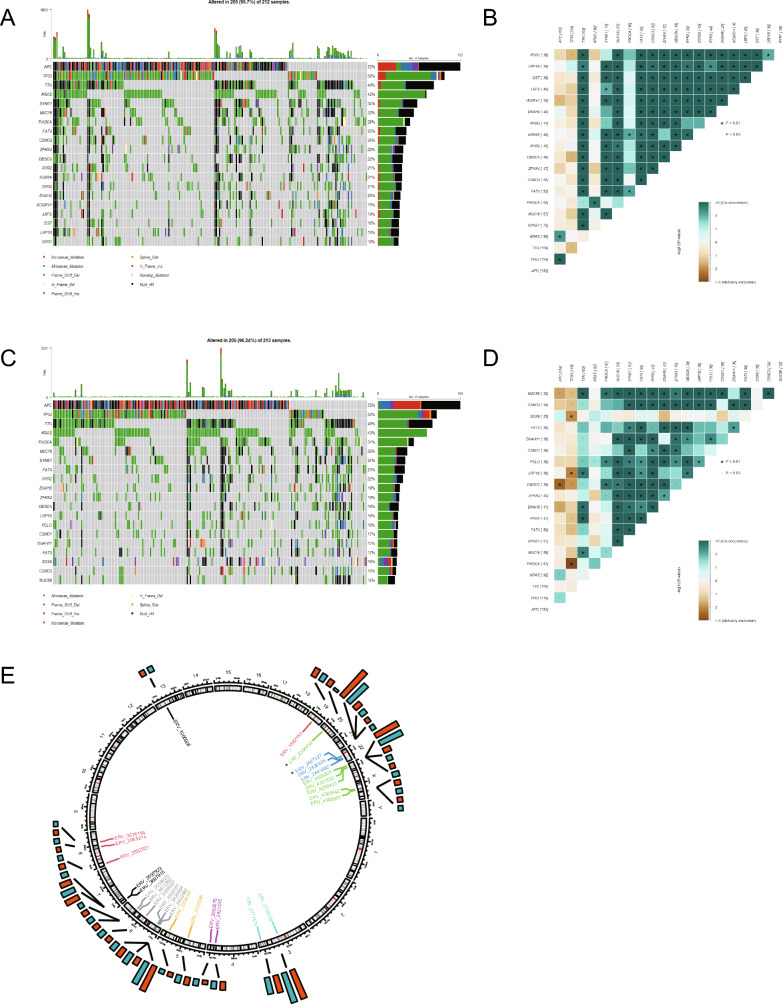
The landscape of genomic alterations between different risk groups. (**A**) Visualization of gene alteration in high KRD score patient. (**B**) Co-occurrence and exclusive mutation analysis of high KRD score patient. (**C**) Visualization of gene alteration in low KRD score patient. (**D**) Co-occurrence and exclusive mutation analysis of low KRD score patient. (**E**) The circos plot showed the position of HERVs, and the barplot showed the proportion of HERVs infection deteced. Statistical differences between groups were calculated by chi-square test. *p < 0.05.

As is widely recognized, the development of cancer is often accompanied by genomic alterations, we identified the top 20 of most frequently mutated genes in high- and low-KRD score group, respectively. As shown in Fig. 7A and C, APC was most altered in both two groups, followed by TP53 and TTN. Interestingly, we found that proportions of KRAS mutation between two groups were very close, while other genes like CSMD3 and USH2A differed, which suggesting that KRD score might reveal some potential differences beyond KRAS mutation. Besides, the co-occurrence and mutually exclusive mutations have been compared between two groups (Fig. 7B, D). A recent research developed a new algorithm to identify viral expression in cancers and applied it to 14 cancer types from TCGA database including COAD. Some recent findings reported that human endogenous retroviruses (HERVs) expression was associated with poor survival rates, so we compared the HERVs expression between two groups. The results indicated that HERVs expression in high-KRD score group patients was higher than low-KRD score group patients, and specifically, one HERV-H member located on chr20 and one HERV-K member located on chr22 were significantly more enriched in high-KRD score group (Fig. 7E).

### Evaluation of TME between high- and low-KRD score group

**Figure 8:**
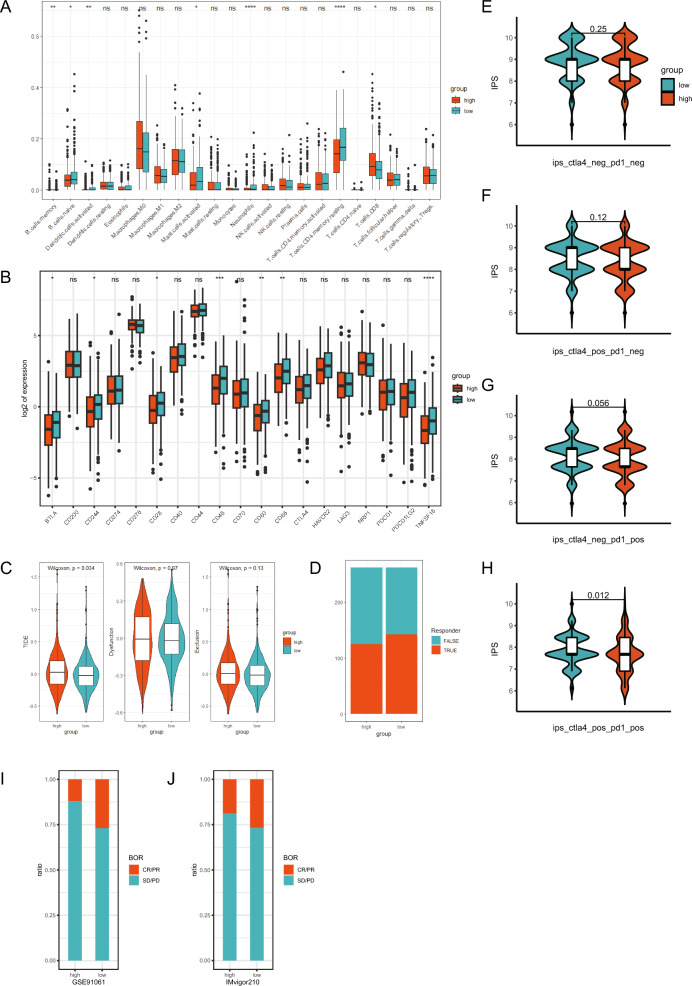
Evaluation of TME between high- and low-KRD score group. (**A**) CIBERSORT calculated the relationsip between immune cell infiltration and KRD score. (**B**) The expression of immune checkpoint genes in high KRD score and low KRD score groups. (**C**) TIDE, T cell dysfunction and exclusion score in high KRD score and low KRD score groups. (**D**) The proportion of responder predicted by TIDE website in high KRD score and low KRD score groups. (**E**–**H**) Differences of IPS with CTLA- and PD1- (**E**), CTLA + and PD1- (**F**), CTLA- and PD1 + (**G**), CTLA- and PD1 + (**H**) between high KRD score and low KRD score groups. (**I**–**J**) Response rate of immunotherapy in two individual cohort (GSE91061 and Imvigor210). Statistical differences between groups were calculated by Wilcox-rank test. *p < 0.05, **p < 0.01, ***p < 0.001, ****p < 0.0001.

Immune cell infiltration was calculated using CIBERSORT algorithm, and the results indicated the probable correlation between many immune cell types and KRD score (Fig. 8A). Further, we explored the relationship between the KRD and expression of immune checkpoint genes (Figure S4A). The results showed that the expression of 19 KRDs was significantly associated with immune checkpoint genes. Low-KRD score group patients had higher expression of immune checkpoint genes including BTLA, CD244, CD28, CD48, CD80, CD86 and TNFSF18, which meant they might benefit from immune checkpoint blockade (ICB) therapy (Fig. 8B).

To further understand the correlation between KRD and efficacy of immunotherapy, we performed Tumor Immune Dysfunction and Exclusion (TIDE) algorithm to calculate each patients’ score. As is shown in Fig. 8C, patients with low-KRD score exhibited significant lower level of TIDE score. The response to anti-PD1 and anti-CTLA4 therapy was predicted by TIDE website. Low-KRD score group patients could benefit more from immunotherapy compared with high-KRD score group (Fig. 8D). Low-KRD score patients also presented higher IPS, indicating higher sensitivity to immunotherapy (Fig. 8E–H). To fully validate our results, two independent immunotherapy cohorts previously published were used to test the efficacy of KRD score (Fig. 8I–J). In both cohorts, low-KRD score groups exhibited higher complete response/partial response (CR/PR) rate as compared with high-KRD score group.

### Drug sensitivity analysis and prediction

**Figure 9:**
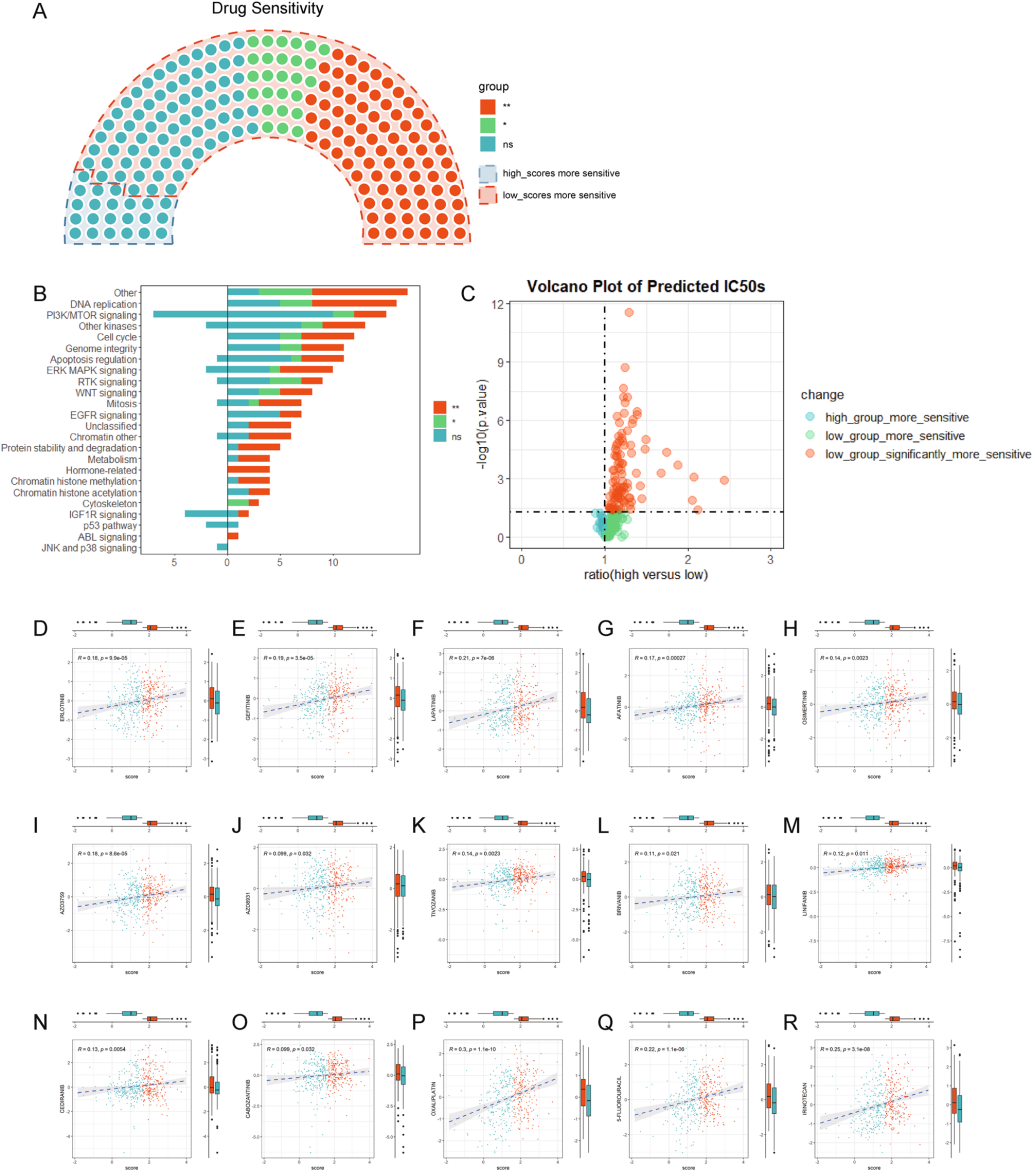
Drug sensitivity analysis and prediction. (**A**) Drug sensitivity analysis between high KRD score and low KRD score groups. Statistical differences between groups were calculated by Wilcox-rank test. *p < 0.05, **p < 0.01. (**B**) The summarize of drug targets. (**C**) The volcano plot of predicted IC50s of drugs. (**D**–**R**) The relationship between KRD score and tested IC50s of drugs including EGFR targeted drugs erlotinib (**D**), gefitinib (**E**), lapatinib (**F**), afatinib (**G**), osimertinib (**H**), AZD3759 (**I**), AZD8931 (**J**), VEGF targeted drugs tivozanib (**K**), brivanib (**L**), linifanib (**M**), cediranib (**N**), cabozantinib (**O**), commonly used chemotherapy drugs oxaliplatin (**P**), 5-fluorouracil (**Q**), irinotecan (**R**).

We performed drug sensitivity analysis to predict IC50 of 198 anti-tumor drugs using pRRophetic package. The results revealed that only 22 drugs presented lower predicted IC50 value in the high-KRD score group patients, but no significant differences were found. However, in the low-KRD score group, better sensitivity was observed regarding other 176 drugs, among which 86 drugs showed significant differences (Fig. 9A). These drugs were further categorized according to their targeting pathway, the results indicated that low-KRD score group patients could benefit from most drug-targeting pathways, including DNA replication, PI3K/MTOR signaling, cell cycle (Fig. 9B–C), etc. Specifically, drugs targeting EGFR signaling all showed lower IC50 in the low-KRD score group patients. As known to all, KRAS mutation patients can hardly benefit from anti-EGFR drugs including cetuximab in that such mutation results in constitutive activation of downstream signaling of EGFR. It was notable that our results indicated that KRD score could predict sensitivity to chemotherapy as well as targeted-therapy, which could not be achieved by KRAS mutation alone. For further validation, we applied the scoring system to cell lines from CCLE and investigated the correlation between KRD scores and standardized drugs IC50s. KRD scores could also predict the drug sensitivity to chemotherapy and targeted-therapy in cell lines data; Commonly used chemotherapy drugs including oxaliplatin, 5-fluorouracil and irinotecan displayed lower IC50 as KRD score decreased (Fig. 9P–R). Drugs targeting EGFR-signaling (Fig. 9D–J) and VEGF-signaling (Fig. 9K–O) showed similar trend.

### Development of a nomogram to predict survival

**Figure 10:**
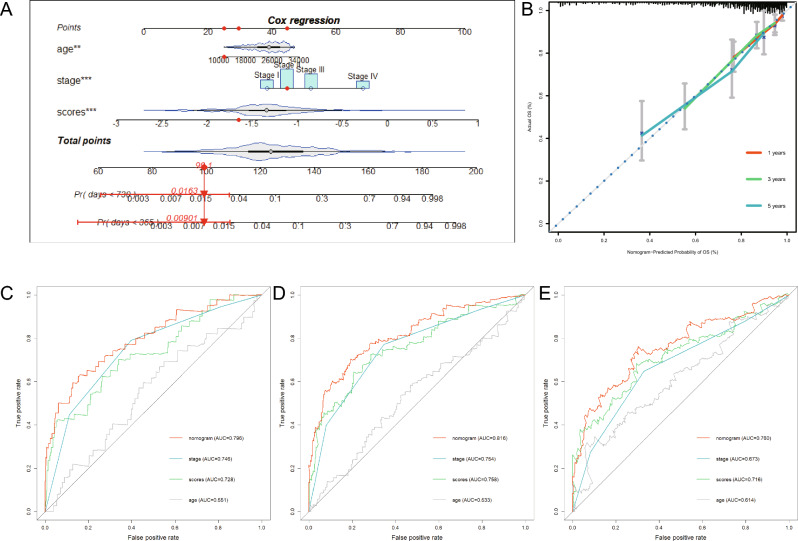
Development of a nomogram to predict survival. (**A**) Construction of nomogram based on KRD score, age and pathology stage. (**B**) Calibration curves of the nomogram in predicting OS of TCGA-COAD patients. ROC curves of the nomogram, KRD score, age and pathology stage in predicting 1 year- (**C**), 3 year- (**D**), 5 year- (**E**) OS of TCGA-COAD patients.

To equip KRD score with a more convenient predictive capacity, a readable nomogram was developed to quantitative measure the 1-, 3-, 5-year OS rates by integrating the KRD score, age and pathological stage (Fig. 10A). The accuracy of nomogram was confirmed by calibration curves (Fig. 10B). ROC curves were constructed to evaluate the performance of nomogram candidates in predicting 1-, 3-, 5-year OS (Fig. 10C–E). The results indicated the nomogram has the best performance in predicting OS in 5-years, compared with age, pathological stage or KRD score alone. Following analysis presented that both stage and scores are independent prognostic factors, whereas age is not.

### SPINK4 affects the biological behaviors of colon cancer cells in vitro

**Figure 11:**
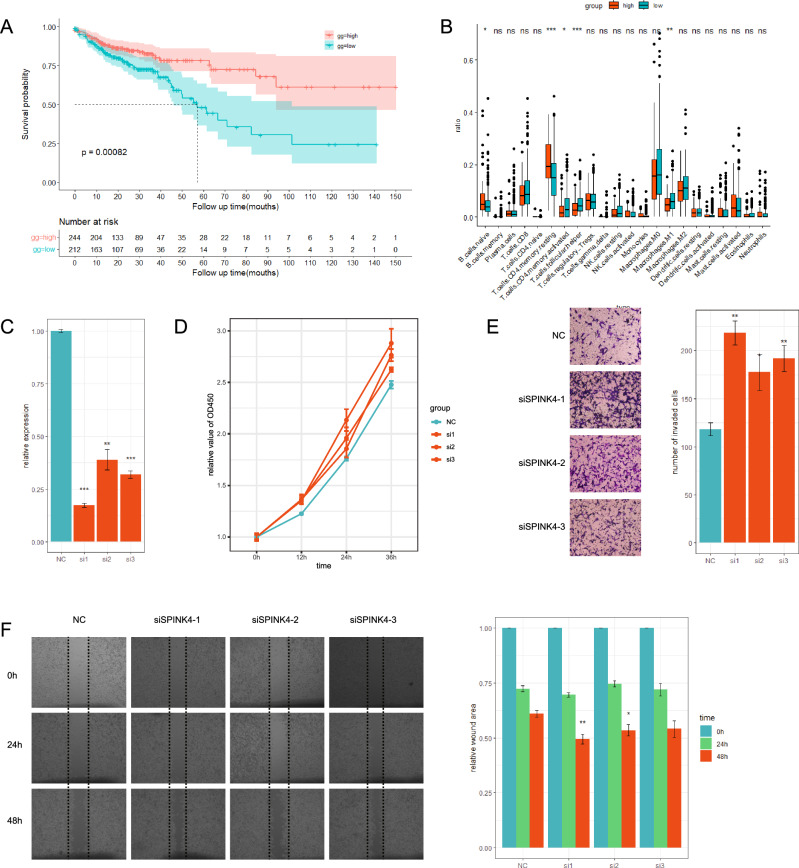
SPINK4 affects the biological behaviors of colon cancer cells in vitro. (**A**) KM survival analysis shows that patient with higher expression level of SPINK4 present a better prognosis compared with low expression group patient. (**B**) CIBERSORT calculated the immune cell infiltration in SPINK4 high expressed group and low expressed group. (**C**) RT-qPCR was performed to detect the dfficiency of SPINK4 knowndown. (**D**) Growth curves of SW48 cells treated with SPINK4 knockdown was performed using cck-8 assay. (**E**) Transwell assay and (**F**) wound healing assay were performed to assess the efficiency of SPINK4 knockdown on the migration of SW48 cells. *p < 0.05, **p < 0.01, ***p < 0.001.

We performed differential expression analysis between high- and low-KRD score groups in the single cell RNAseq database, wherein SPINK4 was significantly upregulated in KRD-low group as shown in Figure S2C. Kaplan–Meier survival analysis revealed that patients with high expression of SPINK4 showed worse overall survivals compared with low SPINK4 group (Fig. 11A). Further analysis showed that the expression of SPINK4 was closely related to the level of immune cell infiltration (Fig. 11B).

Given the biological effects of SPINK4 in colon cancer was still uncertain, we therefore conducted a series of experiments to explore the role SPINK4 played in colon cancer. siRNA for SPINK4 knockdown was designed and transfected into SW48 cells to elucidate how SPINK4 expression affects the biological behaviors. The knockdown efficiency was confirmed by qPCR experiment, and siRNA-1 was the best performer (Fig. 11C). The cck-8 assay was performed to test the cell proliferation, and the knockdown of SPINK4 significantly promoted the proliferation of SW48 cells (Fig. 11D). To investigate whether SPINK4 expression affects cell invasion, transwell assay was conducted. The results indicated that the invasive ability of SW48 was significantly strengthened after the knockdown of SPINK4 by all three siRNAs (Fig. 11E). The wound healing assay also confirmed that knockdown of SPINK4 remarkably raised the migration of SW48 (Fig. 11F). Altogether, our results proved SPINK4 was a tumor suppressor gene by weakening proliferation and migration of tumor cells.

### A pan-cancer analysis of SPINK4

**Figure 12:**
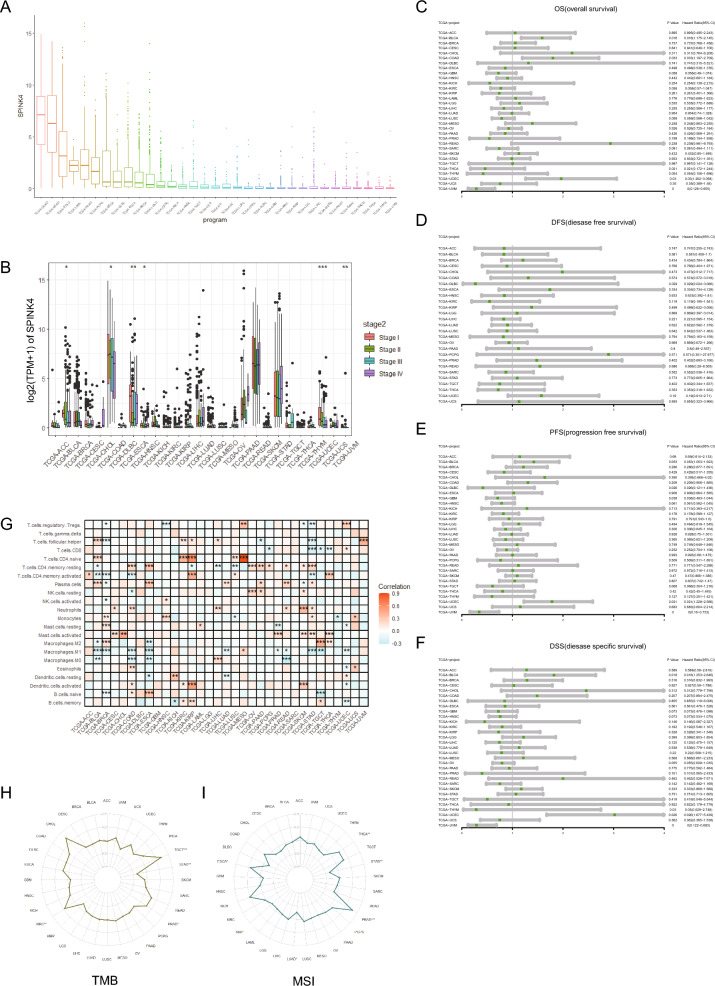
Pan-cancer analysis of SPINK4. (**A**) The expression level of SPINK4 in multiple cancers. (**B**) Correlation analysis between SPINK4 expression and tumor pathology stage. The relationship between SPINK4 expression level and OS (**C**), DFS (**D**), PFS (**E**), DSS (**F**). (**G**) The relationship between SPINK4 expression level and immune cell infiltration. (**H**) Correlation analysis between SPINK4 and tumor mutation burden. (**I**) Analysis of correlation between SPINK4 and Microsatellite instability. *p < 0.05, **p < 0.01, ***p < 0.001.

A pan-cancer analysis showed that SPINK4 was highly expressed in COAD and READ, followed by STAD, LAML, PAAD, PCPG and other cancer types (Fig. 12A). Early stage patients presented much higher expression level of SPINK4 in COAD, UCEC and BLCA (Fig. 12B). We also found that the expression of SPINK4 was significantly correlated with (1) OS of BLCA, COAD, THCA, UCEC and UVM; (2) disease-free survival (DFS) of DLBC; (3) progression-free survival (PFS) of DLBC, GBM, UCEC and UVM; (4) disease specific survival (DSS) of BLCA, THYM, and UVM (Fig. 12C–F). Moreover, we found that SPINK4 was closely related to the level of immune cell infiltration in most tumor types (Fig. 12G), indicating that SPINK4 played an important role in regulating the immune response within the tumor microenvironment. The expression of SPINK4 was significantly correlated with not only the tumor mutation burden of THCA, PRAD, STAD, LUAD and ESCA (Fig. 12H), but also the MSI score of TGCT, STAD, PRAD and KIRC (Fig. 12I). These results suggested that the expression of SPINK4 could be used to evaluate the efficacy of immunotherapy in these tumor types.

## Discussion

Colorectal cancer (CRC) was one of the most prevalent malignant digestive system tumors with leading incidence rate and mortality rate^1^. About 30–50% CRC patients carried somatic KRAS mutation accordingly^14^. However, the prognostic value of KRAS is still under debate.

To our knowledge, no studies have explored predictive value of prognosis and drug response based on a series of well-validated, highly-representative KRAS related signature. Here we first systematically identified and validated the KRAS related signature in COAD by integrating bulk and single cell RNA-seq data. To quantify the overall expression of KRAS related signature, we introduced a novel algorithm namely OE score, which is first developed by Jerby-Arnon, L etc.^31^. This algorithm was developed based on the hypothesis that the measured reads of specific genes were correlated with their real expression accompanied by technical noise. Therefore, it was computed in a way taking accounts of the variation in the signal-to-noise ratio across samples. As a result, OE score was a more reliable system to quantify the expression of a specific gene signature. Applying OE score in both scRNA-seq and bulk RNA-seq data, we developed and validated a series of signature which was closely related with KRAS mutation, and we further confirmed its prognostic value. We compared the KRAS OE score between different pathology stage, and we found that colon cancer patient with distant metastasis presented higher score while no trend revealed with the local progression of tumor. It was in tune with the fact that KRAS mutation was related to bad prognosis in metastatic patients^14^ while uncertain conclusion was observed in localized colon cancer.

Notably, KRAS mutations regulate not only pathway activity within malignant cells, but also cell–cell communications to remodel TME. Accumulating evidence proved that KRAS mutated tumor cells could affect immune cells infiltration, resulting in tumor progression and immune escape^23,24^. One way KRAS mutation facilitated the occurrence and development of tumor was by creating an inflammatory TME^23,37^. Chemokines, a subset of cytokines, are recognized for their pivotal involvement in the complex milieu of cancer-related inflammation, exerting their role through their interactions with their respective receptors^38^. So we explored the role of chemokine and other cytokine based ligand–receptor pairs (LRs) in cell–cell communications from malignant cells to other cell types within TME. Our results firstly indicated distinct LRs profiles between mutation-like and wildtype-like cells, where mutation-like tumor cells were specifically more likely to secret chemokines such as CXCL2/CXCL3 to communicate with other cells. CXCL2 has been reported to promote colon cancer metastasis in vivo, and in vitro stimulation of CXCL2 has been shown to increase colon cancer cell proliferation and migration^39,40^. Besides, CXCL3 has been reported to have potential diagnostic as well as prognostic value. As for other cytokines, TGF-β signaling related LRs were upregulated in mutation-like tumor cell. Our findings were consistent with previous studies, which reported that cytokine and chemokine factors such as IL-6 and CCL5 were involved in the process that KRAS signaling pathway remodeled an inflammatory microenvironment^41–43^. Specifically, the overactivation of KRAS signaling could enhance the excretion of IL-6 resulting in tumor initiation and progression. On the contrary, CCL5, of which expression was elevated by KRAS mutated lung cancer, exhibited antitumor abilities by recruiting T cells to the TME. However, it should be acknowledged that the intrinsic mechanism of these cytokines in remodeling TME in KRAS mutated colon cancer remains unknown, further studies are required.

In addition to acting as a message sender in communications with other cell types, KRAS mutated tumor cells also present some unique characteristics as a receiver. Cancer-associated fibroblasts (CAFs) play an important role in cancer progression by remodeling extracellular matrix (ECM) and extensively interact with cancer cells^44^. Previous work in PDAC showed that KRAS mutation engaged heterotypic fibroblasts, which subsequently instigate reciprocal signaling in tumor cells^45,46^. Our results reflected that mutation-like malignant cells were more likely to receive the messages from fibroblasts. ITGA4, VCAM1 and other ligands were identified as top ligands responsible for the communication from fibroblasts to mutation-like malignant cells. The predicted target genes in malignant cells were mostly enriched in cancer related pathway and several signaling pathways. Previous studies have confirmed that the interaction between CAFs and tumor cells plays a crucial role in tumorigenesis and progression, and targeting CAFs is considered as a highly promising therapeutic intervention^47^. Our findings indicate that mutation-like tumor cells are more susceptible to the direct influence of CAFs, leading to an increase in proliferative potential. The result coincided with a recent study, reporting that VCAM1 secreted by CAFs promoted the tumor growth through AKT and MAPK signaling^48^. Taken together, our results indicated mutation-like malignant cells were more vulnerable to the pro-invasion effect of fibroblasts.

The KRAS related signature was then used to construct a prognostic model we called “KRD score”. Then it was applied to all COAD samples and validated in GEO database. This model was proved to be able to predict the prognosis of colon cancer patients independently and better accuracy was obtained by combining KRD score with other clinical variables.

Then, to further elucidate the capacity of this model in guiding individual treatment strategies, we comprehensively analyzed the differences between high- and low-KRD score group patients. Firstly, we compared the genomic alteration between two groups. In addition to the different mutation subtypes of KRAS, colon cancer patients also possessed other comutations which might contribute to the occurrence and development^18^. Recent research revealed TP53 was the most common comutation in NSCLCs patients with KRAS mutations^49^. Our results revealed that somatic mutations with high frequencies in high-KRD group presented more co-occurrence events compared with low-KRD group. In addition to well-studied gene coding region, other components of the genome remain mysterious. Nearly 9% of human genome is composed of endogenous retroviruses (ERVs)^50^. Here we compared the human endogenous retroviruses (HERVs) load in high- and low-KRD score group patients, and two HERVs were found to be significantly more infected in high-KRD score group tumor tissues. After 30 million years evolution, ERVs now plays unreplaced role in human cells with most functions unknown. It has been proved that ERVs are able to regulate the expression of genes required for normal cell functions by working as transposable elements^51^, which result in copy number variation (CNV). Patients with specific HERVs infections such as HERV-K and HERV-H family, were considered to be correlated with significantly lower overall survival^30,52,53^. In this case, we suppose that CNV, resulted from HERVs infection, was probably associated with the transformation to KRAS mutation-like state of tumor cell.

Then, we explored the predictive value in drug sensitivity of KRD score. As is known to all, KRAS mutated colon cancer acquired resistance to anti-EGFR drugs such as cetuximab due to constitutively active of KRAS protein. What’s worse, KRAS mutated patients usually show poor response to commonly used chemotherapy drugs. Here, the relationship between KRD score and chemotherapy resistance in colon cancer patients was analyzed, and significantly differences in sensitivity to anti-tumor drugs were observed between high- and low-KRD score groups. Interestingly, afatinib, lapatinib and other EGFR targeted drugs showed better therapeutic response in the low-KRD score group. Surprisingly, we found that KRD score was also strongly correlated with the IC50s of VEGF targeted drugs so that this model was able to predict the response to commonly used chemotherapy drugs in colon cancer including 5-Fluorouracil, irinotecan and oxaliplatin, which made KRD score a more robust and widely-applicable model in predicting individual response to different anti-tumor drugs. These results indicated that KRD score helped to distinguish patients subgroup who might benefit more from chemotherapy and targeted therapy.

Finally, we investigated whether KRD score was correlated with immune microenvironment and was thus predictive of the response to immunotherapy. Researches reported that KRAS mediated signaling played an important role in formatting an immunosuppressive TME^54^. A study showed the knockdown of KRAS mutation (G12D) in a poorly immunogenic CRC model could improve the immune response and resulting in tumor regression^55^. Correspondingly, the high-and low-KRD score group patient presented different composition of immune microenvironment. Immune checkpoint genes including CTLA4 and PD-L1 played an important role in regulating the immune response to tumor, and ICB therapy has been widely used to control tumor development. KRAS mutation could upregulate the expression of PD-L1 in tumor cells by different mechanisms including improving the stability of PD-L1 mRNA, promoting ROS production and inducing FGFR1 expression in lung cancer^56,57^. In agreement with evidence above, our results revealed that the KRD score was strongly related to the expression of immune checkpoint genes. Low-KRD score group patients presented higher expression level compared with high-KRD score group, indicating those with lower KRD score might benefit from immune checkpoint inhibitor therapy. Meanwhile low-KRD score group patient showed lower TIDE score, which also support this hypothesis. As for further validation in two independent cohorts, patient with low-KRD score also presented higher response rate following immunotherapy. These findings may draw forth new perspectives for exploring potential mechanisms of KRAS mutation and treatment of colon cancer.

Some clinical trials have shown that targeted therapies greatly prolong progression-free survival with less toxicity compared with standard chemotherapy^4,5^, but unfortunately, patients with KRAS mutation can hardly benefit from neither targeted therapies nor standard chemotherapy^58^. In this case, to explore the potent drug targets for KRAS mutation is a urgent need. However, the relative paucity of biomarkers in mCRC has slowed our progress in this area^59^.

Here, a potential drug target derived from our model was identified. Among 19 candidate genes of KRD score. The expression of SPINK4 was found to be the best independent prognostic index. Serine protease inhibitors Kazal type (SPINK) is one branch of the family of serine protease inhibitors, consisting of a large family of genes with multiple functions. SPINK family members shared a comparable structure known as Kazal type serine protease inhibitor domain^60–62^. The typical Kazal domain composed of 50–60 amino acid residues, with 6 cysteine residues forming three pairs of disulfide bonds for stabilization and a relatively conserved sequence included. SPINKs can regulate serine proteases to prevent the imbalance of protease activity. Some recent studies have reported the relationship between SPINK family and tumors^63–66^. SPINK4 is abundantly expressed in goblet cells, and recent research found that the expression of serum SPINK4 in patients with colon cancer is elevated with high diagnostic value^64^. However, the function of this gene in the pathogenesis of colorectal cancer is undetermined. And no link between the KRAS signaling pathway and SPINK4 could be found in the literature available, warranting further exploration and investigation.

Consequently, we explored the character of SPINK4 in colon cancer microenvironment as well as in other cancer types. The results indicated that the expression of SPINK4 was correlated with immune cell infiltration, which indicated that SPINK4 might participate in the occurrence and development of cancers by regulating the recruitment of immune cells. Then, the functional experiment showed that the silence of SPINK4 promoted the proliferation and migration of cancer cells, suggesting SPINK4 as a tumor suppressor gene. Pan-cancer analysis revealed that SPINK4 expression was significantly associated with survival, clinical stage, immune score, TMB score and MSI in many other cancer types. Altogether, SPINK4 may serve as a potential drug target for the treatment of cancer patients.

It should be acknowledged that our findings still have some limitations. Firstly, this study used mainly online datasets for analysis, and further validation is required with more supplement from clinical data. Moreover, it is still unclear why patients with lower scores of this prognostic model are more likely to benefit from different medications. Besides, the mechanism of SPINK4 in impacting proliferation and migration of tumor cells remains still unclear, so more experiments for confirmation is necessary. In addition, our findings were obtained from a retrospective study and require further validation through a prospective study. With these restrictions, our future work will focus on aspects as listed: (1) conduct animal experiment to verify current findings; (2) design further experiments to explore the underlying molecular mechanisms; (3) collect more clinical information to confirm the effectiveness of our risk model; (4) validate our conclusions with a prospective study. Further optimization is warranted to make this model more applicable and accessible for clinical practice.

## Conclusion

In conclusion, we identified and validated a KRAS related signature and comprehensively explored its role in characterizing genomic alteration, immune microenvironment and drug sensitivity of colon cancer. We proved that KRAS related signature might serve as a predictor for individual prognosis and response to treatments. The findings hold the potential for translational applications within the clinical domain, offering the prospect of prognostic modeling for patient outcomes and serving as a guiding compass for the administration of pharmaceutical interventions. The expression of SPINK4 was a independent prognostic predictor, and the knockdown of SPINK4 enhanced the proliferation and migration of colon cancer cells. This gene presents a convincing prospect as a promising drug target warranting further exploration and rigorous investigation.

### Supplementary Information

Supplementary Figure 1.

Supplementary Information 2.

Supplementary Table 3.

## Data Availability

The public datasets were downloaded and analyzed in this study, which can be found in GEO data repository and included the accession numbers as follows: GSE91061, GSE166555, GSE39582. Any reasonable requests for access to source code used in this article will be considered. Such proposals should be submitted to the corresponding author.

## Abbreviations

AUC: Area under the ROC curve
CAFs: Cancer-associated fibroblasts
CCLE: Broad Institute Cancer Cell Line Encyclopedia
CDF: Cumulative distribution function
CR: Complete response
DEGs: Differentially expressed genes
DFS: Disease-free survival
dMMR: MisMatch repair-deficient
DSS: Disease specific survival
ECM: Extracellular matrix
EGFR: Epidermal growth factor receptor
FC: Fold change
GEO: Gene Expression Omnibus
GSVA: Gene set variation analysis
HERVs: Human endogenous retroviruses
ICB: Immune checkpoint blockade
IL: Interleukin
IPS: Immunophenoscore
KRDs: KRAS related differentially expressed genes
LRs: Ligand–receptor pairs
mCRC: Metastatic colorectal cancer
MSigDB: Molecular signatures database
MSI-H: MicroSatellite instability-high
OE score: Overall expression score
OS: Overall survival
PFS: Progression-free survival
PR: Partial response
RAS: Rat sarcoma viral oncogene family
SPINK: Serine protease inhibitors Kazal type
ssGSEA: Single sample gene set enrichment analysis
TCGA: The Cancer Genome Atlas
TGF-β: Transforming growth factor-β
TMB: Tumor mutation berden
TME: Tumor microenvironment
TPM: Transcripts per kilobase million
UMAP: Uniform manifold approximation and projection
VEGF: Vascular endothelial growth factor
