# Supplementary material for: Leveraging a KRAS-based signature to predict the prognosis and drug sensitivity of colon cancer and identifying SPINK4 as a new biomarker
Source: Sci Rep. 2023 Dec 14;13:22230. doi: 10.1038/s41598-023-48768-0 (PMC10721872; doi:10.1038/s41598-023-48768-0)
Supplement: Supplementary file 1 — Supplementary Figure 1. [file 41598_2023_48768_MOESM1_ESM.docx]

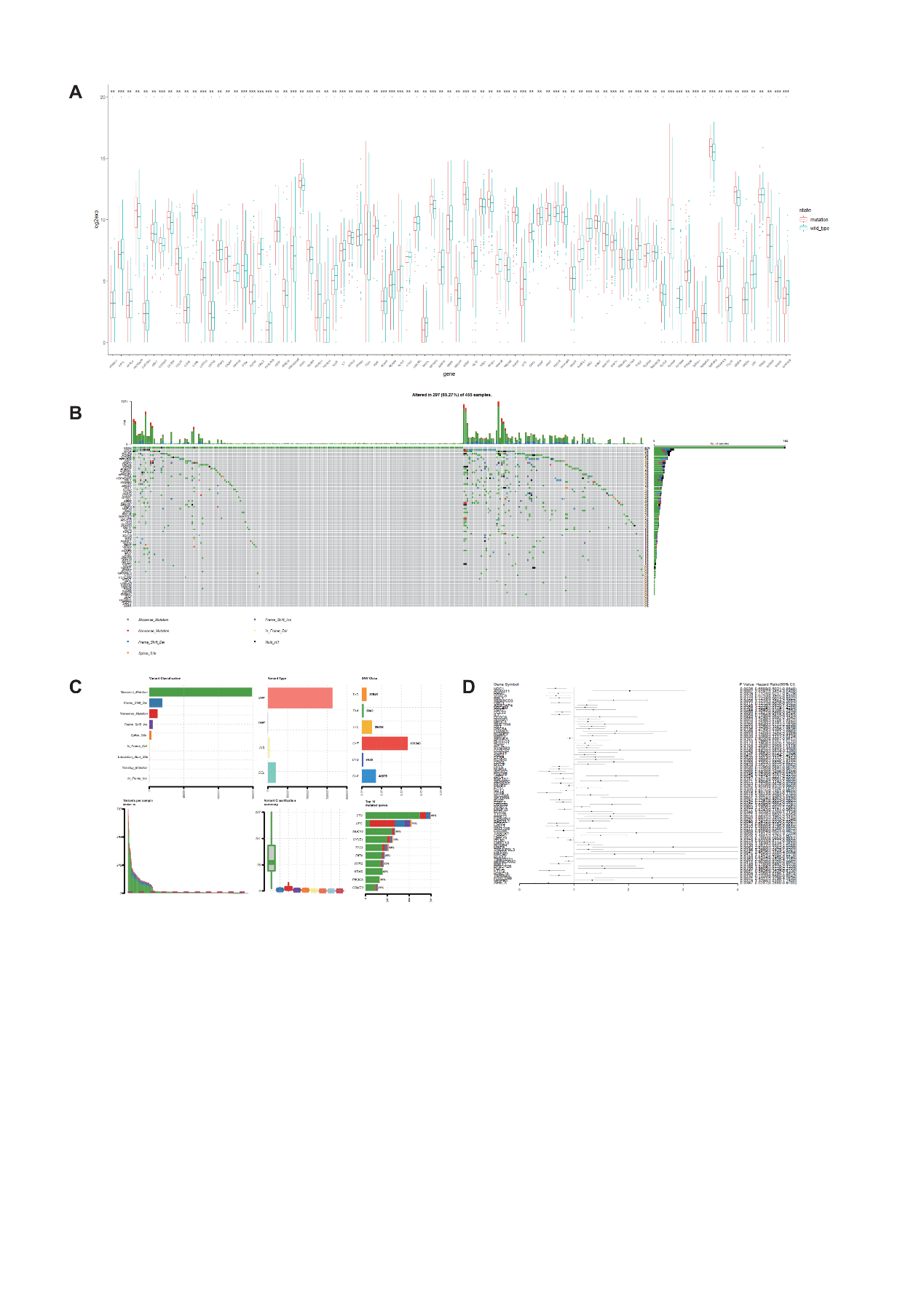
Supple 1

Summarize of 80 KRGs in KRAS mutation and KRAS wildtype patients. (A) The expression level of 80 KRGs between KRASmt and KRASwt group. (B) The genetic alteration profile of 80 KRGs. (C) The summerize of genetic alteration in all colon cancer patient. (D) The forest plot presented the result of univariate cox regression analysis of 80 KRGs.


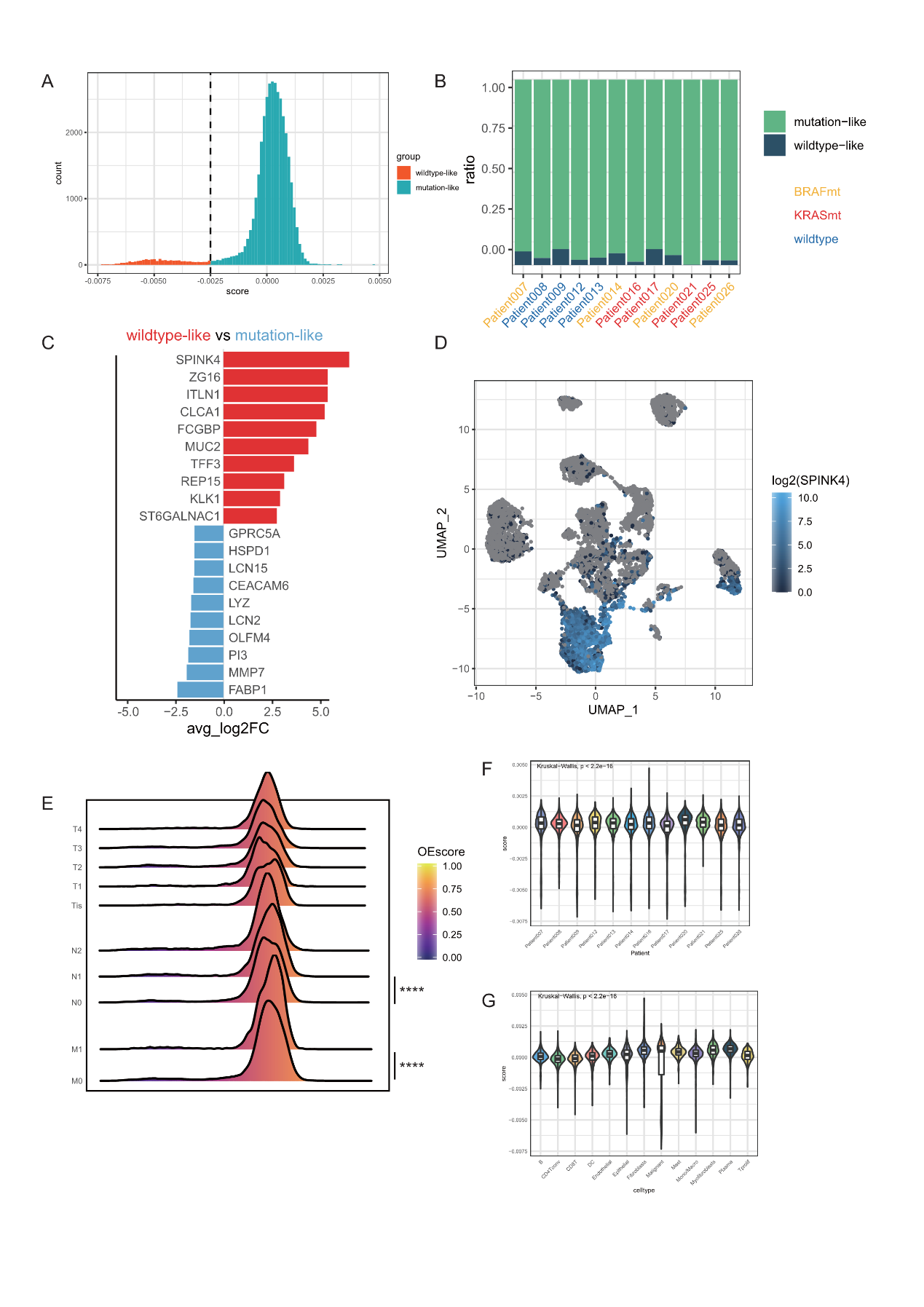
Supple 2

KRAS OE score at single cell level. (A) The distribution of KRAS OE score of all cells. (B) The proportion of mutation-like and wildtype-like cells in each patients. (C) The top differentially expressed genes between wildtype-like cells and mutation-like cells. (D) The expression of SPINK4 gene in different malignant cell clusters. (E) Ridge plot of KRAS OE score distribution in different pathological stage. (F) Violin plot of KRAS OE score in each patient. (G) Violin plot of KRAS OE score in each cell types. Statistical differences between groups were calculated by Wilcox-rank test. ****p<0.0001.


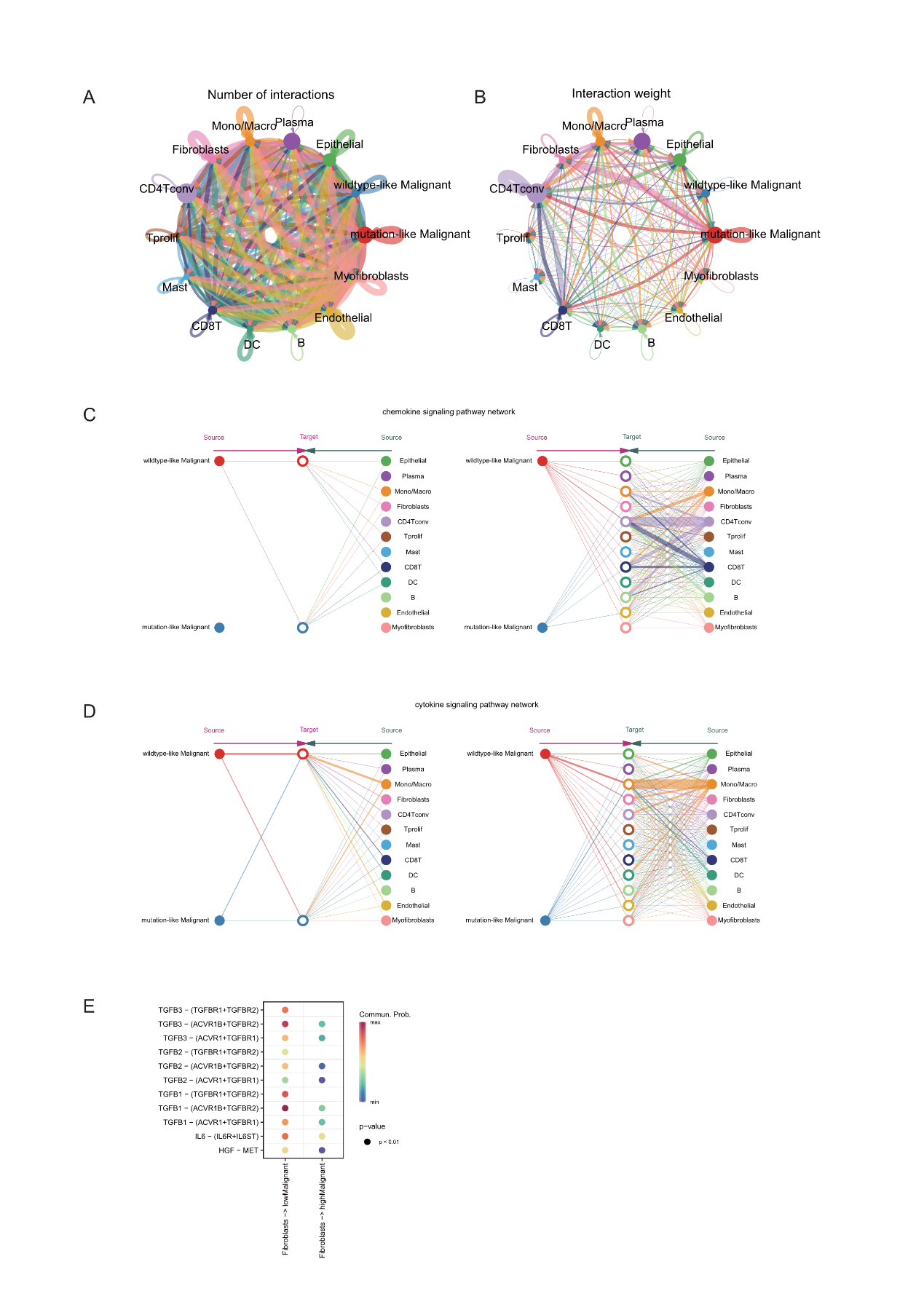


Supple 3

Overall cell-cell interaction and chemokines/cytokines related pathways. (A) The number of interactions over all cell types. (B) The weight of interactions over all cell types. (C-D) The hierarchical plot of pathways including chemokines (C) and other cytokines (D). (E) Interactions between fibroblasts and malignant cells through ligand-receptor (LRs).


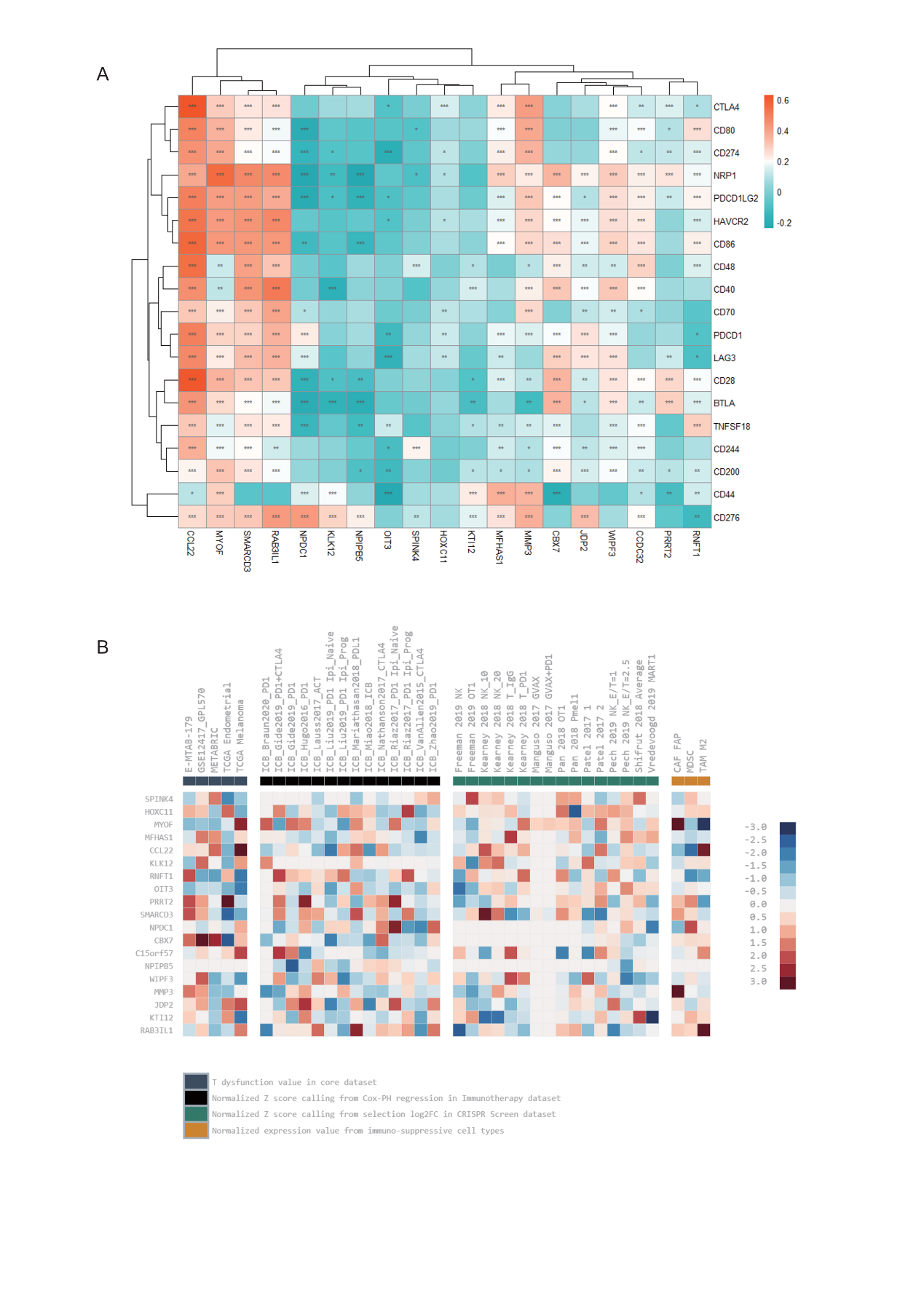


Supple 4

The relationship between KRD score and immunotherapy. (A) The heatmap of relationsips between 19 KRDs and common immune checkpoint genes. (B) Enrichment of 19 KRDs in T cell dysfunction level, ICB response outcome, phenotypes in genetic screens and celltypes promoting T cell exclusion.
