# Supplementary material for: Leveraging a KRAS-based signature to predict the prognosis and drug sensitivity of colon cancer and identifying SPINK4 as a new biomarker
Source: Sci Rep. 2023 Dec 14;13:22230. doi: 10.1038/s41598-023-48768-0 (PMC10721872; doi:10.1038/s41598-023-48768-0)
Supplement: Supplementary file 2 — Supplementary Information 2. [file 41598_2023_48768_MOESM2_ESM.docx]

The sequences of the SPINK4 siRNA were as follows:

5’GCACUGAUGGGCUCACAUAUA3’ (si1);

5’GGCUCACAUAUACGAAUGAAU3’ (si2);

5’GGCAUGGAGAGGAUAUGACAU3’ (si3).

The sequence of primers were listed as follow:

SPINK4

forward strand CAGTGGGTAATCGCCCTGG;

reverse strand CACAGATGGGCATTCTTGAGAAA.
