# Supplementary material for: Leveraging a KRAS-based signature to predict the prognosis and drug sensitivity of colon cancer and identifying SPINK4 as a new biomarker
Source: Sci Rep. 2023 Dec 14;13:22230. doi: 10.1038/s41598-023-48768-0 (PMC10721872; doi:10.1038/s41598-023-48768-0)
Supplement: Supplementary file 3 — Supplementary Table 3. [file 41598_2023_48768_MOESM3_ESM.docx]

**Supplementary table 1**

The identified 80 KRAS-related Differentially Expressed Genes. Gene symbols, adjusted p value of KM analysis and univariate cox regression analysis are provided.

| **symbol** | **KM_adjusted_p** | **cox_adjusted_p** |
| --- | --- | --- |
| NDC1 | 0.021935929 | 0.002644427 |
| ADAM11 | 0.036149477 | 0.000759352 |
| RBM7 | 0.036358148 | 0.032945859 |
| VDAC3 | 0.004269273 | 0.01200621 |
| RBL1 | 0.031553199 | 0.01235708 |
| SMARCD3 | 0.00984484 | 0.000680605 |
| ORC1 | 0.000386475 | 0.011770683 |
| ARHGAP4 | 0.044122802 | 0.004571762 |
| NRCAM | 0.046956841 | 0.030580751 |
| CBX7 | 0.021977387 | 0.024484215 |
| CCL22 | 0.008604951 | 0.009984584 |
| IL7 | 0.01166424 | 0.006452763 |
| CCDC9 | 0.022623909 | 0.044405815 |
| ISYNA1 | 0.011447085 | 0.005348128 |
| NPDC1 | 0.025690684 | 0.032476484 |
| SEPTIN4 | 0.021407933 | 0.0205393 |
| UST | 0.02105844 | 0.001997981 |
| UNC5A | 0.017627475 | 0.019642491 |
| LMAN2L | 0.008941358 | 0.006977617 |
| KCNIP3 | 0.009901564 | 0.001098475 |
| WIPF3 | 0.028983349 | 0.002093263 |
| SPINK4 | 0.001194344 | 0.010769387 |
| HOXC13 | 0.000576653 | 0.041018991 |
| HOXC11 | 0.045913056 | 0.017315279 |
| AIF1L | 0.019075099 | 0.010443586 |
| SYNGR3 | 0.047753379 | 0.013649693 |
| CCDC32 | 0.001499222 | 0.024568783 |
| SOX15 | 0.046803792 | 0.012674006 |
| SHC2 | 0.01369852 | 0.043122175 |
| DTNA | 0.032455175 | 0.003916028 |
| KCNH3 | 0.009996613 | 0.036071686 |
| CDK9 | 0.026541978 | 0.025967052 |
| MYOF | 0.014156195 | 0.042919621 |
| OIT3 | 0.042453925 | 0.003696385 |
| NUP54 | 0.011190183 | 0.028893273 |
| INTS12 | 0.009320911 | 0.006888317 |
| POC1B | 0.029065292 | 0.034690908 |
| JDP2 | 0.00214767 | 0.002427238 |
| RNF157 | 0.000280288 | 0.018721626 |
| SEMA6C | 0.021824449 | 0.007169208 |
| MFHAS1 | 0.039977248 | 0.032334004 |
| MMP3 | 0.033294515 | 0.028226664 |
| FZD7 | 0.024223356 | 0.015885119 |
| NAT2 | 0.023516658 | 0.030333611 |
| CD1B | 0.002794404 | 0.001625201 |
| SLC2A6 | 0.030853048 | 0.021546094 |
| SPTBN4 | 0.030853014 | 0.000196924 |
| ICOS | 0.0118287 | 0.024262747 |
| F2RL2 | 0.018964082 | 0.014200112 |
| DIPK1B | 0.038578133 | 0.00014107 |
| WDR72 | 0.040328386 | 0.046314943 |
| MMP10 | 0.039046799 | 0.007763168 |
| ELFN2 | 0.042662933 | 0.034829195 |
| TTC16 | 0.010619894 | 0.027116176 |
| PRRT2 | 0.032807051 | 0.002542355 |
| CASKIN1 | 0.042192911 | 0.029732639 |
| RAB3IL1 | 0.001027334 | 0.004889967 |
| PGM2 | 0.011466939 | 0.007904574 |
| HINT1 | 0.016844452 | 0.043325294 |
| WNT10B | 0.010128562 | 0.021145035 |
| CXCR6 | 0.0329932 | 0.046987734 |
| TAS1R1 | 0.011075437 | 0.00688037 |
| CSRP2 | 0.026182664 | 0.004874587 |
| UBE2N | 0.046576228 | 0.042419504 |
| ITLN1 | 0.038291578 | 0.027949021 |
| CHST13 | 0.022746407 | 0.033206589 |
| HOXB4 | 0.01532451 | 0.013005206 |
| TNFAIP8L3 | 0.02524158 | 0.014652253 |
| CNTN2 | 0.011246014 | 0.004790141 |
| MROH7 | 0.038288687 | 0.047454248 |
| KLK12 | 0.040838577 | 0.01892339 |
| TMEM221 | 0.036555277 | 0.036357332 |
| HEPACAM2 | 0.014563502 | 0.041559328 |
| RNFT1 | 0.006114258 | 0.014995607 |
| PPP1R26 | 0.015227272 | 0.016894094 |
| PHF2 | 0.003174086 | 0.019925583 |
| KTI12 | 0.010252729 | 0.005182669 |
| TMSB4X | 0.000567417 | 0.013236488 |
| NPIPB5 | 0.041554771 | 0.037920905 |
| APELA | 0.001208616 | 0.039747735 |

**Supplementary table 2**

The detailed ligand-receptors (LRs) table. Wilcoxon Signed Rank Test was used to compare the LRs’ ‘CommunProb’ calculated using ‘cellchat’ between mutation-like cells and wildtype-like cells.

| LRs | W | pvalue | qvalue | role |
| --- | --- | --- | --- | --- |
| LTA_TNFRSF14 | 196 | 4.99E-08 | 1.83E-05 | sender |
| TGFB3_TGFBR1_TGFBR2 | 194 | 1.99E-07 | 7.30E-05 | sender |
| GAS6_AXL | 194 | 1.99E-07 | 7.30E-05 | sender |
| JAG1_NOTCH3 | 194 | 1.99E-07 | 7.30E-05 | sender |
| TGFB1_ACVR1B_TGFBR21 | 194 | 1.99E-07 | 9.09E-05 | receiver |
| TGFB2_ACVR1B_TGFBR21 | 194 | 1.99E-07 | 9.09E-05 | receiver |
| TGFB3_ACVR1B_TGFBR21 | 194 | 1.99E-07 | 9.09E-05 | receiver |
| JAG1_NOTCH11 | 194 | 1.99E-07 | 9.09E-05 | receiver |
| JAG1_NOTCH1 | 193 | 3.49E-07 | 0.000127 | sender |
| CD46_JAG11 | 193 | 3.49E-07 | 0.000158 | receiver |
| TGFB3_ACVR1_TGFBR1 | 191 | 9.47E-07 | 0.000343 | sender |
| EREG_EGFR | 196 | 1.69E-06 | 0.000608 | sender |
| EREG_EGFR_ERBB2 | 196 | 1.69E-06 | 0.000608 | sender |
| EREG_ERBB4 | 196 | 1.69E-06 | 0.000608 | sender |
| EREG_ERBB2_ERBB4 | 196 | 1.69E-06 | 0.000608 | sender |
| CXCL2_CXCR2 | 196 | 1.69E-06 | 0.000608 | sender |
| CXCL3_CXCR2 | 196 | 1.69E-06 | 0.000608 | sender |
| NAMPT_INSR | 196 | 1.69E-06 | 0.000608 | sender |
| NAMPT_ITGA5_ITGB1 | 196 | 1.69E-06 | 0.000608 | sender |
| MDK_ITGA4_ITGB1 | 196 | 1.69E-06 | 0.000608 | sender |
| MDK_ITGA6_ITGB1 | 196 | 1.69E-06 | 0.000608 | sender |
| MDK_LRP1 | 196 | 1.69E-06 | 0.000608 | sender |
| MDK_NCL | 196 | 1.69E-06 | 0.000608 | sender |
| MDK_ALK | 196 | 1.69E-06 | 0.000608 | sender |
| SEMA3C_NRP1_PLXNA2 | 196 | 1.69E-06 | 0.000608 | sender |
| SEMA3C_NRP2_PLXNA2 | 196 | 1.69E-06 | 0.000608 | sender |
| LAMA3_ITGA1_ITGB1 | 196 | 1.69E-06 | 0.000608 | sender |
| LAMB1_ITGA1_ITGB1 | 196 | 1.69E-06 | 0.000608 | sender |
| LAMC2_ITGA1_ITGB1 | 196 | 1.69E-06 | 0.000608 | sender |
| LAMA3_ITGA2_ITGB1 | 196 | 1.69E-06 | 0.000608 | sender |
| LAMB1_ITGA2_ITGB1 | 196 | 1.69E-06 | 0.000608 | sender |
| LAMC2_ITGA2_ITGB1 | 196 | 1.69E-06 | 0.000608 | sender |
| LAMA3_ITGA6_ITGB1 | 196 | 1.69E-06 | 0.000608 | sender |
| LAMB1_ITGA6_ITGB1 | 196 | 1.69E-06 | 0.000608 | sender |
| LAMC2_ITGA6_ITGB1 | 196 | 1.69E-06 | 0.000608 | sender |
| LAMA3_ITGA7_ITGB1 | 196 | 1.69E-06 | 0.000608 | sender |
| LAMB1_ITGA7_ITGB1 | 196 | 1.69E-06 | 0.000608 | sender |
| LAMC2_ITGA7_ITGB1 | 196 | 1.69E-06 | 0.000608 | sender |
| LAMA3_ITGA6_ITGB4 | 196 | 1.69E-06 | 0.000608 | sender |
| LAMB1_ITGA6_ITGB4 | 196 | 1.69E-06 | 0.000608 | sender |
| LAMC2_ITGA6_ITGB4 | 196 | 1.69E-06 | 0.000608 | sender |
| LAMA3_CD44 | 196 | 1.69E-06 | 0.000608 | sender |
| LAMB1_CD44 | 196 | 1.69E-06 | 0.000608 | sender |
| LAMC2_CD44 | 196 | 1.69E-06 | 0.000608 | sender |
| EFNB1_EPHA4 | 196 | 1.69E-06 | 0.000608 | sender |
| EFNB1_EPHB2 | 196 | 1.69E-06 | 0.000608 | sender |
| EFNB1_EPHB4 | 196 | 1.69E-06 | 0.000608 | sender |
| DLL1_NOTCH1 | 0 | 1.69E-06 | 0.000608 | sender |
| DLL1_NOTCH3 | 0 | 1.69E-06 | 0.000608 | sender |
| TGFB1_TGFBR1_TGFBR21 | 190 | 1.50E-06 | 0.000675 | receiver |
| TGFB2_TGFBR1_TGFBR21 | 190 | 1.50E-06 | 0.000675 | receiver |
| TGFB3_ACVR1B_TGFBR2 | 189 | 2.24E-06 | 0.000725 | sender |
| PRSS3_F2RL11 | 196 | 1.69E-06 | 0.000757 | receiver |
| F2_F2RL11 | 196 | 1.69E-06 | 0.000757 | receiver |
| HSPG2_DAG11 | 196 | 1.69E-06 | 0.000757 | receiver |
| CDH1_ITGAE_ITGB71 | 196 | 1.69E-06 | 0.000757 | receiver |
| CD1D_CEACAM51 | 196 | 1.69E-06 | 0.000757 | receiver |
| CD8A_CEACAM51 | 196 | 1.69E-06 | 0.000757 | receiver |
| EFNB2_EPHB31 | 196 | 1.69E-06 | 0.000757 | receiver |
| EFNB3_EPHB31 | 196 | 1.69E-06 | 0.000757 | receiver |
| FGF9_FGFR1 | 188 | 3.34E-06 | 0.001076 | sender |
| TGFB3_TGFBR1_TGFBR21 | 188 | 3.34E-06 | 0.001473 | receiver |
| TGFB1_ACVR1_TGFBR11 | 188 | 3.34E-06 | 0.001473 | receiver |
| TGFB2_ACVR1_TGFBR11 | 188 | 3.34E-06 | 0.001473 | receiver |
| THBS1_CD47 | 186 | 6.93E-06 | 0.002224 | sender |
| WNT3A_FZD8_LRP61 | 185 | 9.72E-06 | 0.004258 | receiver |
| IGF2_IGF1R1 | 185 | 9.72E-06 | 0.004258 | receiver |
| FGF9_FGFR2 | 184 | 1.36E-05 | 0.004339 | sender |
| LTA_LTB_LTBR | 184 | 1.36E-05 | 0.004339 | sender |
| FN1_ITGA4_ITGB7 | 183 | 1.86E-05 | 0.005913 | sender |
| LTA_TNFRSF1A | 182 | 2.53E-05 | 0.008028 | sender |
| FN1_ITGA4_ITGB1 | 182 | 2.53E-05 | 0.008028 | sender |
| L1CAM_ITGA4_ITGB71 | 182 | 2.53E-05 | 0.011042 | receiver |
| TGFB3_ACVR1_TGFBR11 | 181 | 3.40E-05 | 0.01479 | receiver |
| IL1A_IL1R21 | 15 | 3.40E-05 | 0.01479 | receiver |
| CD99_CD99 | 186.5 | 5.26E-05 | 0.016556 | sender |
| LTA_TNFRSF1B | 179 | 5.97E-05 | 0.018754 | sender |
| KLK3_NGFR | 179 | 5.97E-05 | 0.018754 | sender |
| HGF_MET | 179 | 5.97E-05 | 0.018754 | sender |
| HGF_MET1 | 180 | 4.53E-05 | 0.019623 | receiver |
| CD99_CD991 | 186.5 | 5.26E-05 | 0.022705 | receiver |
| BMP4_BMPR1B_ACVR2B | 178 | 7.82E-05 | 0.024327 | sender |
| TGFA_EGFR1 | 179 | 5.97E-05 | 0.025742 | receiver |
| FCER2A_ITGAX_ITGB21 | 179 | 5.97E-05 | 0.025742 | receiver |
| ADGRE5_CD551 | 175 | 6.65E-05 | 0.028532 | receiver |
| MSTN_ACVR1B_ACVR2A1 | 177 | 0.000101 | 0.043422 | receiver |
| FCER2A_ITGAM_ITGB21 | 177 | 0.000101 | 0.043422 | receiver |
| GDF15_TGFBR2 | 175 | 0.000167 | 0.051867 | sender |
| TNFSF14_TNFRSF14 | 175 | 0.000167 | 0.051867 | sender |
| CNTN2_L1CAM | 175 | 0.000167 | 0.051867 | sender |
| MSTN_ACVR1B_ACVR2B1 | 176 | 0.000131 | 0.055729 | receiver |
| WNT3A_FZD8_LRP51 | 174 | 0.000213 | 0.090431 | receiver |
| BMP4_BMPR1B_ACVR2A | 172 | 0.000338 | 0.103617 | sender |
| GDF5_BMPR1B_ACVR2B | 172 | 0.000338 | 0.103617 | sender |
| TGFA_EGFR_ERBB21 | 173 | 0.000269 | 0.113915 | receiver |
| LTA_TNFRSF1A1 | 173 | 0.000269 | 0.113915 | receiver |
| BTLA_TNFRSF14 | 175.5 | 0.000402 | 0.122755 | sender |
| EGF_EGFR_ERBB2 | 171 | 0.000421 | 0.128067 | sender |
| KLK3_NTRK1 | 171 | 0.000421 | 0.128067 | sender |
| ICAM1_ITGAX_ITGB2 | 171 | 0.000421 | 0.128067 | sender |
| TNFSF14_TNFRSF141 | 172 | 0.000338 | 0.142432 | receiver |
| COL1A1_ITGA2_ITGB1 | 170 | 0.000523 | 0.157506 | sender |
| BTLA_TNFRSF141 | 175.5 | 0.000402 | 0.169442 | receiver |
| CSF3_CSF3R | 173.5 | 0.000568 | 0.17034 | sender |
| L1CAM_L1CAM | 173.5 | 0.000568 | 0.17034 | sender |
| EGF_EGFR1 | 171 | 0.000421 | 0.176934 | receiver |
| JAG1_NOTCH31 | 171 | 0.000421 | 0.176934 | receiver |
| WNT3A_FZD8_LRP6 | 169 | 0.000646 | 0.192572 | sender |
| COL1A1_ITGA1_ITGB1 | 169 | 0.000646 | 0.192572 | sender |
| FN1_ITGA8_ITGB1 | 169 | 0.000646 | 0.192572 | sender |
| GP1BA_ITGAM_ITGB21 | 170 | 0.000523 | 0.218729 | receiver |
| TGFB1_TGFBR1_TGFBR2 | 168 | 0.000794 | 0.234343 | sender |
| TGFB2_TGFBR1_TGFBR2 | 168 | 0.000794 | 0.234343 | sender |
| FN1_ITGA5_ITGB1 | 168 | 0.000794 | 0.234343 | sender |
| CSF3_CSF3R1 | 173.5 | 0.000568 | 0.236772 | receiver |
| L1CAM_L1CAM1 | 173.5 | 0.000568 | 0.236772 | receiver |
| GH1_GHR1 | 169 | 0.000646 | 0.26818 | receiver |
| GH_GHR1 | 169 | 0.000646 | 0.26818 | receiver |
| CNTN2_L1CAM1 | 169 | 0.000646 | 0.26818 | receiver |
| CNTN2_CNTN2 | 170.5 | 0.000937 | 0.273706 | sender |
| BMP2_BMPR1B_ACVR2B | 167 | 0.000971 | 0.282668 | sender |
| GDF5_BMPR1B_ACVR2A | 167 | 0.000971 | 0.282668 | sender |
| FCER2A_ITGAX_ITGB2 | 167 | 0.000971 | 0.282668 | sender |
| AREG_EGFR_ERBB2 | 166 | 0.001183 | 0.340618 | sender |
| COL1A1_CD44 | 166 | 0.001183 | 0.340618 | sender |
| MIF_CD74_CXCR2 | 168.5 | 0.001296 | 0.370767 | sender |
| CNTN2_CNTN21 | 170.5 | 0.000937 | 0.386188 | receiver |
| MSTN_TGFBR1_ACVR2B | 165 | 0.001433 | 0.408411 | sender |
| EGF_EGFR | 165 | 0.001433 | 0.408411 | sender |
| GH_PRLR | 165 | 0.001433 | 0.408411 | sender |
| SEMA4D_PLXNB1 | 165 | 0.001433 | 0.408411 | sender |
| MSTN_TGFBR1_ACVR2A1 | 166 | 0.001183 | 0.486091 | receiver |
| INHBA_ACVR1B_ACVR2A1 | 166 | 0.001183 | 0.486091 | receiver |
| FGF1_FGFR41 | 166 | 0.001183 | 0.486091 | receiver |
| IL1B_IL1R21 | 30 | 0.001183 | 0.486091 | receiver |
| OCLN_OCLN | 166.5 | 0.001779 | 0.499853 | sender |
| FGF9_FGFR3 | 163 | 0.002078 | 0.581712 | sender |
| GH1_GHR | 163 | 0.002078 | 0.581712 | sender |
| NGF_NGFR | 163 | 0.002078 | 0.581712 | sender |
| GH_GHR | 163 | 0.002078 | 0.581712 | sender |
| THY1_ITGAX_ITGB2 | 163 | 0.002078 | 0.581712 | sender |
| MSTN_TGFBR1_ACVR2B1 | 165 | 0.001433 | 0.58324 | receiver |
| FGF2_FGFR41 | 165 | 0.001433 | 0.58324 | receiver |
| GH_PRLR1 | 165 | 0.001433 | 0.58324 | receiver |
| FCER2A_CR21 | 165 | 0.001433 | 0.58324 | receiver |
| FGF9_FGFR4 | 162 | 0.002486 | 0.683733 | sender |
| FN1_CD44 | 162 | 0.002486 | 0.683733 | sender |
| INHBA_ACVR1B_ACVR2B1 | 164 | 0.001729 | 0.696912 | receiver |
| OCLN_OCLN1 | 166.5 | 0.001779 | 0.715093 | receiver |
| PRL_PRLR | 163.5 | 0.002817 | 0.769102 | sender |
| FLT3L_FLT3 | 163.5 | 0.002817 | 0.769102 | sender |
| WNT3A_FZD8_LRP5 | 161 | 0.002963 | 0.803003 | sender |
| TGFA_EGFR_ERBB2 | 161 | 0.002963 | 0.803003 | sender |
| AREG_EGFR | 161 | 0.002963 | 0.803003 | sender |
| IGF2_IGF2R | 161 | 0.002963 | 0.803003 | sender |
| ICAM1_ITGAM_ITGB2 | 161 | 0.002963 | 0.803003 | sender |
| L1CAM_ITGA4_ITGB7 | 161 | 0.002963 | 0.803003 | sender |
| EGF_EGFR_ERBB21 | 163 | 0.002078 | 0.833095 | receiver |
| IGF2_IGF2R1 | 163 | 0.002078 | 0.833095 | receiver |
| NGF_NGFR1 | 163 | 0.002078 | 0.833095 | receiver |
| SEMA4D_PLXNB11 | 163 | 0.002078 | 0.833095 | receiver |
| ALCAM_CD6 | 147 | 0.003392 | 0.898845 | sender |
| CD6_ALCAM | 147 | 0.003392 | 0.898845 | sender |
| TGFB1_ACVR1B_TGFBR2 | 160 | 0.003519 | 0.925377 | sender |
| TGFB2_ACVR1B_TGFBR2 | 160 | 0.003519 | 0.925377 | sender |
| TGFB1_ACVR1_TGFBR1 | 160 | 0.003519 | 0.925377 | sender |
| TGFB2_ACVR1_TGFBR1 | 160 | 0.003519 | 0.925377 | sender |
| IL1B_IL1R1_IL1RAP | 36 | 0.003519 | 0.925377 | sender |
| C3_ITGAX_ITGB2 | 160 | 0.003519 | 0.925377 | sender |
| BDNF_NTRK2 | 160 | 0.003519 | 0.925377 | sender |
| EFNB2_EPHB2 | 160 | 0.003519 | 0.925377 | sender |
| GDNF_GFRA1 | 161.5 | 0.00379 | 0.966546 | sender |
| LIF_LIFR_IL6ST1 | 162 | 0.002486 | 0.987062 | receiver |
| BMP2_BMPR1B_ACVR2A | 159 | 0.004162 | 1 | sender |
| BMP2_BMPR1B_BMPR2 | 145 | 0.031025 | 1 | sender |
| BMP4_BMPR1B_BMPR2 | 152 | 0.012248 | 1 | sender |
| GDF5_BMPR1B_BMPR2 | 147 | 0.024124 | 1 | sender |
| MSTN_ACVR1B_ACVR2B | 149 | 0.018554 | 1 | sender |
| MSTN_ACVR1B_ACVR2A | 143 | 0.039482 | 1 | sender |
| MSTN_TGFBR1_ACVR2A | 155 | 0.007866 | 1 | sender |
| INHBA_ACVR1B_ACVR2A | 145 | 0.031025 | 1 | sender |
| INHBA_ACVR1B_ACVR2B | 149 | 0.018554 | 1 | sender |
| WNT3A_FZD1_LRP5 | 98.5 | 1 | 1 | sender |
| WNT3A_FZD4_LRP5 | 98.5 | 1 | 1 | sender |
| WNT3A_FZD5_LRP5 | 102.5 | 0.797931 | 1 | sender |
| WNT3A_FZD6_LRP5 | 98.5 | 1 | 1 | sender |
| WNT3A_FZD7_LRP5 | 98.5 | 1 | 1 | sender |
| WNT3A_FZD1_LRP6 | 98.5 | 1 | 1 | sender |
| WNT3A_FZD4_LRP6 | 98.5 | 1 | 1 | sender |
| WNT3A_FZD5_LRP6 | 102.5 | 0.797931 | 1 | sender |
| WNT3A_FZD6_LRP6 | 98.5 | 1 | 1 | sender |
| WNT3A_FZD7_LRP6 | 98.5 | 1 | 1 | sender |
| TGFA_EGFR | 158 | 0.004906 | 1 | sender |
| HBEGF_EGFR | 133 | 0.113919 | 1 | sender |
| HBEGF_EGFR_ERBB2 | 138 | 0.069048 | 1 | sender |
| HBEGF_ERBB4 | 140 | 0.055617 | 1 | sender |
| HBEGF_ERBB2_ERBB4 | 143 | 0.039482 | 1 | sender |
| NRG1_ERBB3 | 159 | 0.004162 | 1 | sender |
| NRG1_ERBB2_ERBB3 | 157 | 0.005761 | 1 | sender |
| NRG1_ERBB4 | 158 | 0.004906 | 1 | sender |
| NRG1_ERBB2_ERBB4 | 155 | 0.007866 | 1 | sender |
| FGF1_FGFR1 | 155 | 0.007866 | 1 | sender |
| FGF1_FGFR2 | 137 | 0.076664 | 1 | sender |
| FGF1_FGFR3 | 134 | 0.103523 | 1 | sender |
| FGF1_FGFR4 | 131 | 0.137068 | 1 | sender |
| FGF2_FGFR1 | 155 | 0.007866 | 1 | sender |
| FGF2_FGFR2 | 137 | 0.076664 | 1 | sender |
| FGF2_FGFR3 | 134 | 0.103523 | 1 | sender |
| FGF2_FGFR4 | 131 | 0.137068 | 1 | sender |
| FGF7_FGFR1 | 116 | 0.427387 | 1 | sender |
| FGF7_FGFR2 | 113 | 0.511239 | 1 | sender |
| FGF3_FGFR1 | 155 | 0.007866 | 1 | sender |
| FGF3_FGFR2 | 136 | 0.084928 | 1 | sender |
| FGF10_FGFR1 | 138 | 0.069048 | 1 | sender |
| FGF10_FGFR2 | 134.5 | 0.098058 | 1 | sender |
| PDGFA_PDGFRA | 124 | 0.24563 | 1 | sender |
| PDGFA_PDGFRB | 122 | 0.285176 | 1 | sender |
| PDGFB_PDGFRA | 137 | 0.076664 | 1 | sender |
| PDGFB_PDGFRB | 139 | 0.062042 | 1 | sender |
| PDGFC_PDGFRA | 133 | 0.113919 | 1 | sender |
| VEGFA_VEGFR1 | 156 | 0.006743 | 1 | sender |
| VEGFA_VEGFR2 | 156 | 0.006743 | 1 | sender |
| VEGFB_VEGFR1 | 155 | 0.007866 | 1 | sender |
| VEGFC_VEGFR3 | 152 | 0.012248 | 1 | sender |
| VEGFC_VEGFR2 | 149 | 0.018554 | 1 | sender |
| PGF_VEGFR1 | 155 | 0.007866 | 1 | sender |
| VEGFA_VEGFR1R2 | 155 | 0.007866 | 1 | sender |
| VEGFC_VEGFR2R3 | 148 | 0.021187 | 1 | sender |
| IGF1_IGF1R | 152 | 0.012248 | 1 | sender |
| IGF2_IGF1R | 150 | 0.016203 | 1 | sender |
| IGF1_ITGA6_ITGB4 | 146 | 0.027396 | 1 | sender |
| IGF2_ITGA6_ITGB4 | 143 | 0.039482 | 1 | sender |
| CCL5_CCR1 | 127 | 0.193579 | 1 | sender |
| CCL3_CCR1 | 125 | 0.227321 | 1 | sender |
| CCL5_CCR3 | 125.5 | 0.214695 | 1 | sender |
| CCL11_CCR3 | 125.5 | 0.214695 | 1 | sender |
| CCL4_CCR5 | 130 | 0.149881 | 1 | sender |
| CCL5_CCR5 | 133 | 0.113919 | 1 | sender |
| CCL3_CCR5 | 130 | 0.149881 | 1 | sender |
| CCL20_CCR6 | 105 | 0.353111 | 1 | sender |
| CCL5_ACKR1 | 98.5 | 1 | 1 | sender |
| CXCL2_ACKR1 | 105 | 0.353111 | 1 | sender |
| CXCL3_ACKR1 | 105 | 0.353111 | 1 | sender |
| CCL11_ACKR1 | 98.5 | 1 | 1 | sender |
| CXCL12_CXCR4 | 135.5 | 0.089075 | 1 | sender |
| CXCL12_ACKR3 | 100 | 0.909855 | 1 | sender |
| CXCL16_CXCR6 | 105 | 0.353111 | 1 | sender |
| MIF_CD74_CXCR4 | 140.5 | 0.053599 | 1 | sender |
| MIF_CD74_CD44 | 154.5 | 0.01007 | 1 | sender |
| MIF_ACKR3 | 100 | 0.909855 | 1 | sender |
| IL3_IL3RA_CSF2RB | 136 | 0.084928 | 1 | sender |
| CSF2_CSF2RA_CSF2RB | 135.5 | 0.089075 | 1 | sender |
| IL4_IL4R | 119 | 0.35189 | 1 | sender |
| IL4_IL4R_IL13RA1 | 116 | 0.427387 | 1 | sender |
| IL4_IL4R_IL13RA2 | 121 | 0.306426 | 1 | sender |
| IL13_IL4R_IL13RA1 | 117 | 0.401263 | 1 | sender |
| IL13_IL4R_IL13RA2 | 121 | 0.306426 | 1 | sender |
| IL13_IL13RA1 | 119 | 0.35189 | 1 | sender |
| IL13_IL13RA2 | 124 | 0.24563 | 1 | sender |
| IL6_IL6R_IL6ST | 137 | 0.076664 | 1 | sender |
| CNTF_CNTFR_LIFR | 151 | 0.014108 | 1 | sender |
| LIF_LIFR_IL6ST | 150 | 0.016203 | 1 | sender |
| OSM_LIFR_IL6ST | 150 | 0.016203 | 1 | sender |
| IL1A_IL1R1_IL1RAP | 130 | 0.149881 | 1 | sender |
| IL1A_IL1R2 | 128 | 0.178112 | 1 | sender |
| IL1B_IL1R2 | 44 | 0.012248 | 1 | sender |
| IL34_CSF1R | 149 | 0.018554 | 1 | sender |
| CSF1_CSF1R | 149.5 | 0.019096 | 1 | sender |
| TNFSF14_LTBR | 146 | 0.027396 | 1 | sender |
| FASL_FAS | 150 | 0.016203 | 1 | sender |
| TNFSF15_TNFRSF25 | 98.5 | 1 | 1 | sender |
| TNFSF10_TNFRSF10B | 127 | 0.193579 | 1 | sender |
| TNFSF13B_TNFRSF17 | 124.5 | 0.232164 | 1 | sender |
| TNFSF13B_TNFRSF13B | 126.5 | 0.198195 | 1 | sender |
| TNFSF13B_TNFRSF13C | 128.5 | 0.168012 | 1 | sender |
| CD40LG_ITGA5_ITGB1 | 150 | 0.016203 | 1 | sender |
| CD40LG_ITGAM_ITGB2 | 155 | 0.007866 | 1 | sender |
| MDK_SDC1 | 126 | 0.037919 | 1 | sender |
| MDK_SDC2 | 119 | 0.079464 | 1 | sender |
| MDK_SDC4 | 126 | 0.037919 | 1 | sender |
| MDK_PTPRZ1 | 105 | 0.353111 | 1 | sender |
| C3_C3AR1 | 98.5 | 1 | 1 | sender |
| C3_CR2 | 152.5 | 0.013083 | 1 | sender |
| C3_ITGAM_ITGB2 | 151 | 0.014108 | 1 | sender |
| C4A_C3AR1 | 98.5 | 1 | 1 | sender |
| C4A_CR2 | 145 | 0.031025 | 1 | sender |
| HC_C5AR1 | 98.5 | 1 | 1 | sender |
| PRSS3_F2R | 151 | 0.014108 | 1 | sender |
| F2_F2R | 148 | 0.021187 | 1 | sender |
| PRSS3_F2RL1 | 100 | 0.909855 | 1 | sender |
| F2_F2RL1 | 100 | 0.909855 | 1 | sender |
| PRSS3_F2RL3 | 98.5 | 1 | 1 | sender |
| F2_F2RL3 | 98.5 | 1 | 1 | sender |
| PRSS3_PARD3 | 142 | 0.044369 | 1 | sender |
| F2_PARD3 | 141 | 0.049736 | 1 | sender |
| NPPC_NPR2 | 147.5 | 0.024338 | 1 | sender |
| KITL_KIT | 153.5 | 0.011489 | 1 | sender |
| NGF_NTRK1 | 154 | 0.009146 | 1 | sender |
| SEMA3B_NRP1_PLXNA2 | 126 | 0.209978 | 1 | sender |
| SEMA3B_NRP1_PLXNA3 | 99 | 0.969895 | 1 | sender |
| SEMA3C_NRP1_PLXNA3 | 112 | 0.164915 | 1 | sender |
| SEMA3B_NRP2_PLXNA2 | 128 | 0.178112 | 1 | sender |
| SEMA3B_NRP2_PLXNA3 | 100 | 0.909855 | 1 | sender |
| SEMA3C_NRP2_PLXNA3 | 112 | 0.164915 | 1 | sender |
| SEMA3C_NRP1_NRP2 | 119 | 0.079464 | 1 | sender |
| SEMA3C_PLXND1 | 119 | 0.079464 | 1 | sender |
| ADM_CALCRL | 98.5 | 1 | 1 | sender |
| ANXA1_FPR1 | 98.5 | 1 | 1 | sender |
| GAS6_MERTK | 98.5 | 1 | 1 | sender |
| GRN_SORT1 | 123 | 0.26491 | 1 | sender |
| LGALS9_CD45 | 102 | 0.866972 | 1 | sender |
| PSAP_GPR37 | 155 | 0.007866 | 1 | sender |
| LGALS9_HAVCR2 | 97.5 | 1 | 1 | sender |
| LGALS9_CD44 | 102 | 0.874287 | 1 | sender |
| FN1_ITGA3_ITGB1 | 98.5 | 1 | 1 | sender |
| LAMB3_ITGA1_ITGB1 | 152 | 0.012248 | 1 | sender |
| LAMB3_ITGA2_ITGB1 | 147 | 0.024124 | 1 | sender |
| LAMA3_ITGA3_ITGB1 | 105 | 0.353111 | 1 | sender |
| LAMB1_ITGA3_ITGB1 | 105 | 0.353111 | 1 | sender |
| LAMB3_ITGA3_ITGB1 | 98.5 | 1 | 1 | sender |
| LAMC2_ITGA3_ITGB1 | 105 | 0.353111 | 1 | sender |
| RELN_ITGA3_ITGB1 | 98.5 | 1 | 1 | sender |
| THBS1_ITGA3_ITGB1 | 98.5 | 1 | 1 | sender |
| COL1A1_ITGA3_ITGB1 | 98.5 | 1 | 1 | sender |
| LAMB3_ITGA6_ITGB1 | 139 | 0.062042 | 1 | sender |
| LAMB3_ITGA7_ITGB1 | 146 | 0.027396 | 1 | sender |
| COL1A1_ITGA9_ITGB1 | 98.5 | 1 | 1 | sender |
| LAMA3_ITGA9_ITGB1 | 105 | 0.353111 | 1 | sender |
| LAMB1_ITGA9_ITGB1 | 105 | 0.353111 | 1 | sender |
| LAMB3_ITGA9_ITGB1 | 98.5 | 1 | 1 | sender |
| LAMC2_ITGA9_ITGB1 | 105 | 0.353111 | 1 | sender |
| COL1A1_ITGA11_ITGB1 | 98.5 | 1 | 1 | sender |
| FN1_ITGAV_ITGB1 | 152 | 0.012248 | 1 | sender |
| LAMB3_ITGA6_ITGB4 | 133 | 0.113919 | 1 | sender |
| LAMB3_CD44 | 145 | 0.031025 | 1 | sender |
| COL1A1_SDC1 | 106 | 0.665664 | 1 | sender |
| FN1_SDC1 | 105 | 0.708038 | 1 | sender |
| THBS1_SDC1 | 106 | 0.665664 | 1 | sender |
| COL1A1_SDC4 | 105 | 0.708038 | 1 | sender |
| FN1_SDC4 | 105 | 0.708038 | 1 | sender |
| THBS1_SDC4 | 105 | 0.708038 | 1 | sender |
| THBS1_CD36 | 98.5 | 1 | 1 | sender |
| AGRN_DAG1 | 126 | 0.037919 | 1 | sender |
| HSPG2_DAG1 | 106 | 0.665664 | 1 | sender |
| LAMA3_DAG1 | 126 | 0.037919 | 1 | sender |
| LAMB1_DAG1 | 126 | 0.037919 | 1 | sender |
| LAMB3_DAG1 | 103 | 0.795432 | 1 | sender |
| LAMC2_DAG1 | 126 | 0.037919 | 1 | sender |
| ADGRE5_CD55 | 123 | 0.24098 | 1 | sender |
| APP_CD74 | 138.5 | 0.066039 | 1 | sender |
| CD22_PTPRC | 121 | 0.281559 | 1 | sender |
| FCER2A_ITGAM_ITGB2 | 155 | 0.007866 | 1 | sender |
| FCER2A_CR2 | 156 | 0.006743 | 1 | sender |
| CD40LG_CD40 | 141.5 | 0.048152 | 1 | sender |
| CD46_JAG1 | 152 | 0.012248 | 1 | sender |
| CD80_CD28 | 149 | 0.018554 | 1 | sender |
| CD80_CTLA4 | 142 | 0.044369 | 1 | sender |
| CD86_CD28 | 149 | 0.018554 | 1 | sender |
| CD86_CTLA4 | 143 | 0.039482 | 1 | sender |
| CD99_PILRA | 98.5 | 1 | 1 | sender |
| CDH2_CDH2 | 140.5 | 0.053599 | 1 | sender |
| CDH1_CDH1 | 147.5 | 0.024338 | 1 | sender |
| CDH1_ITGA1_ITGB1 | 159 | 0.004162 | 1 | sender |
| CDH1_ITGAE_ITGB7 | 120.5 | 0.279988 | 1 | sender |
| CDH1_KLRG1 | 148 | 0.021187 | 1 | sender |
| CDH5_CDH5 | 139.5 | 0.05955 | 1 | sender |
| CD1D_CEACAM5 | 100 | 0.909855 | 1 | sender |
| CD8A_CEACAM5 | 100 | 0.909855 | 1 | sender |
| CEACAM1_CEACAM5 | 112 | 0.164915 | 1 | sender |
| DSC2_DSG2 | 100.5 | 0.898139 | 1 | sender |
| EFNA1_EPHA1 | 132 | 0.125092 | 1 | sender |
| EFNA1_EPHA2 | 139 | 0.062042 | 1 | sender |
| EFNA1_EPHA3 | 140 | 0.055617 | 1 | sender |
| EFNA1_EPHA4 | 133 | 0.113919 | 1 | sender |
| EFNA1_EPHA7 | 136 | 0.084928 | 1 | sender |
| EFNA2_EPHA1 | 128 | 0.178112 | 1 | sender |
| EFNA2_EPHA2 | 133 | 0.113919 | 1 | sender |
| EFNA2_EPHA3 | 136 | 0.084928 | 1 | sender |
| EFNA2_EPHA4 | 129 | 0.163552 | 1 | sender |
| EFNA2_EPHA7 | 133 | 0.113919 | 1 | sender |
| EFNA4_EPHA1 | 136 | 0.084928 | 1 | sender |
| EFNA4_EPHA2 | 142 | 0.044369 | 1 | sender |
| EFNA4_EPHA3 | 142 | 0.044369 | 1 | sender |
| EFNA4_EPHA4 | 134 | 0.103523 | 1 | sender |
| EFNA4_EPHA7 | 139 | 0.062042 | 1 | sender |
| EFNA5_EPHA1 | 126 | 0.209978 | 1 | sender |
| EFNA5_EPHA2 | 130 | 0.149881 | 1 | sender |
| EFNA5_EPHA3 | 135.5 | 0.089075 | 1 | sender |
| EFNA5_EPHA4 | 127 | 0.193579 | 1 | sender |
| EFNA5_EPHA7 | 128 | 0.178112 | 1 | sender |
| EFNA5_EPHB2 | 151 | 0.014108 | 1 | sender |
| EFNB1_EPHB3 | 105 | 0.353111 | 1 | sender |
| EFNB2_EPHA4 | 131 | 0.137068 | 1 | sender |
| EFNB2_EPHB3 | 98.5 | 1 | 1 | sender |
| EFNB2_EPHB4 | 131 | 0.137068 | 1 | sender |
| EFNB3_EPHA4 | 128 | 0.178112 | 1 | sender |
| EFNB3_EPHB2 | 151 | 0.014108 | 1 | sender |
| EFNB3_EPHB3 | 98.5 | 1 | 1 | sender |
| EFNB3_EPHB4 | 128 | 0.178112 | 1 | sender |
| GP1BA_ITGAM_ITGB2 | 158 | 0.004906 | 1 | sender |
| ICAM1_ITGAL_ITGB2 | 151 | 0.014108 | 1 | sender |
| ICAM1_ITGAL | 147 | 0.024124 | 1 | sender |
| ITGB2_ICAM1 | 156 | 0.006743 | 1 | sender |
| ITGB2_ICAM2 | 100 | 0.909855 | 1 | sender |
| JAM1_ITGAL_ITGB2 | 136 | 0.084928 | 1 | sender |
| F11R_F11R | 106 | 0.687621 | 1 | sender |
| F11R_JAM2 | 98.5 | 1 | 1 | sender |
| F11R_JAM3 | 102.5 | 0.797931 | 1 | sender |
| HLA-A_CD8A | 148 | 0.021187 | 1 | sender |
| HLA-B_CD8A | 148 | 0.021187 | 1 | sender |
| HLA-C_CD8A | 148 | 0.021187 | 1 | sender |
| HLA-E_CD8A | 139 | 0.062042 | 1 | sender |
| HLA-F_CD8A | 148 | 0.021187 | 1 | sender |
| HLA-C_KIR2DL3 | 149.5 | 0.019096 | 1 | sender |
| HLA-E_KLRC1 | 99 | 0.969895 | 1 | sender |
| HLA-F_LILRB1 | 99 | 0.969895 | 1 | sender |
| HLA-E_CD94:NKG2A | 99 | 0.969895 | 1 | sender |
| HLA-DRB1_CD4 | 123.5 | 0.250618 | 1 | sender |
| MPZL1_MPZL1 | 136.5 | 0.080767 | 1 | sender |
| JAG1_NOTCH2 | 106 | 0.665664 | 1 | sender |
| DLL1_NOTCH2 | 70 | 0.037919 | 1 | sender |
| SEMA4D_PLXNB2 | 147 | 0.024124 | 1 | sender |
| SEMA4D_CD72 | 98.5 | 1 | 1 | sender |
| SEMA5A_PLXNA3 | 112 | 0.164915 | 1 | sender |
| THY1_ITGAM_ITGB2 | 152 | 0.012248 | 1 | sender |
| ITGA4_ITGB1_VCAM1 | 98.5 | 1 | 1 | sender |
| ITGA4_ITGB7_VCAM1 | 98.5 | 1 | 1 | sender |
| BMP2_BMPR1B_ACVR2A1 | 144 | 0.035045 | 1 | receiver |
| BMP2_BMPR1B_ACVR2B1 | 144 | 0.035045 | 1 | receiver |
| BMP2_BMPR1B_BMPR21 | 145 | 0.031025 | 1 | receiver |
| BMP4_BMPR1B_ACVR2A1 | 141 | 0.049736 | 1 | receiver |
| BMP4_BMPR1B_ACVR2B1 | 141 | 0.049736 | 1 | receiver |
| BMP4_BMPR1B_BMPR21 | 143 | 0.039482 | 1 | receiver |
| GDF5_BMPR1B_ACVR2A1 | 157 | 0.005761 | 1 | receiver |
| GDF5_BMPR1B_ACVR2B1 | 155 | 0.007866 | 1 | receiver |
| GDF5_BMPR1B_BMPR21 | 161 | 0.002963 | 1 | receiver |
| GDF15_TGFBR21 | 100 | 0.909855 | 1 | receiver |
| GDNF_GFRA11 | 161.5 | 0.00379 | 1 | receiver |
| INHBB_ACVR1B_ACVR2A | 98.5 | 1 | 1 | receiver |
| INHBB_ACVR1B_ACVR2B | 98.5 | 1 | 1 | receiver |
| INHBABB_ACVR1B_ACVR2A | 98.5 | 1 | 1 | receiver |
| INHBABB_ACVR1B_ACVR2B | 98.5 | 1 | 1 | receiver |
| WNT2B_FZD5_LRP5 | 98.5 | 1 | 1 | receiver |
| WNT2B_FZD8_LRP5 | 98.5 | 1 | 1 | receiver |
| WNT3A_FZD5_LRP51 | 136 | 0.084928 | 1 | receiver |
| WNT2B_FZD5_LRP6 | 98.5 | 1 | 1 | receiver |
| WNT2B_FZD8_LRP6 | 98.5 | 1 | 1 | receiver |
| WNT3A_FZD5_LRP61 | 158 | 0.004906 | 1 | receiver |
| WNT5A_FZD5 | 98.5 | 1 | 1 | receiver |
| WNT5A_FZD8 | 98.5 | 1 | 1 | receiver |
| AREG_EGFR1 | 142 | 0.043166 | 1 | receiver |
| AREG_EGFR_ERBB21 | 140 | 0.053715 | 1 | receiver |
| HBEGF_EGFR1 | 115 | 0.400711 | 1 | receiver |
| HBEGF_EGFR_ERBB21 | 113 | 0.460216 | 1 | receiver |
| HBEGF_ERBB41 | 110 | 0.558077 | 1 | receiver |
| HBEGF_ERBB2_ERBB41 | 111 | 0.524366 | 1 | receiver |
| EREG_EGFR1 | 100 | 0.909855 | 1 | receiver |
| EREG_EGFR_ERBB21 | 100 | 0.909855 | 1 | receiver |
| EREG_ERBB41 | 100 | 0.909855 | 1 | receiver |
| EREG_ERBB2_ERBB41 | 100 | 0.909855 | 1 | receiver |
| NRG1_ERBB31 | 157 | 0.005761 | 1 | receiver |
| NRG1_ERBB2_ERBB31 | 160 | 0.003519 | 1 | receiver |
| NRG1_ERBB41 | 159 | 0.004162 | 1 | receiver |
| NRG1_ERBB2_ERBB41 | 161 | 0.002963 | 1 | receiver |
| FGF1_FGFR11 | 134 | 0.103523 | 1 | receiver |
| FGF1_FGFR21 | 127 | 0.193579 | 1 | receiver |
| FGF1_FGFR31 | 138 | 0.069048 | 1 | receiver |
| FGF2_FGFR11 | 134 | 0.103523 | 1 | receiver |
| FGF2_FGFR21 | 127 | 0.193579 | 1 | receiver |
| FGF2_FGFR31 | 141 | 0.049736 | 1 | receiver |
| FGF7_FGFR11 | 133 | 0.113919 | 1 | receiver |
| FGF7_FGFR21 | 129 | 0.163552 | 1 | receiver |
| FGF3_FGFR11 | 132 | 0.125092 | 1 | receiver |
| FGF3_FGFR21 | 128 | 0.178112 | 1 | receiver |
| FGF10_FGFR11 | 135 | 0.093868 | 1 | receiver |
| FGF10_FGFR21 | 134.5 | 0.098058 | 1 | receiver |
| FGF9_FGFR11 | 128 | 0.178112 | 1 | receiver |
| FGF9_FGFR21 | 121 | 0.306426 | 1 | receiver |
| FGF9_FGFR31 | 134 | 0.103523 | 1 | receiver |
| FGF9_FGFR41 | 158 | 0.004906 | 1 | receiver |
| PDGFA_PDGFRA1 | 128 | 0.178112 | 1 | receiver |
| PDGFA_PDGFRB1 | 135 | 0.093868 | 1 | receiver |
| PDGFB_PDGFRA1 | 130 | 0.149881 | 1 | receiver |
| PDGFB_PDGFRB1 | 141 | 0.049736 | 1 | receiver |
| PDGFC_PDGFRA1 | 132 | 0.125092 | 1 | receiver |
| VEGFA_VEGFR11 | 134 | 0.103523 | 1 | receiver |
| VEGFA_VEGFR21 | 137 | 0.076664 | 1 | receiver |
| VEGFB_VEGFR11 | 149 | 0.018554 | 1 | receiver |
| VEGFC_VEGFR31 | 142 | 0.044369 | 1 | receiver |
| VEGFC_VEGFR21 | 152 | 0.012248 | 1 | receiver |
| PGF_VEGFR11 | 143 | 0.039482 | 1 | receiver |
| VEGFA_VEGFR1R21 | 135 | 0.093868 | 1 | receiver |
| VEGFC_VEGFR2R31 | 148 | 0.021187 | 1 | receiver |
| IGF1_IGF1R1 | 157 | 0.005761 | 1 | receiver |
| IGF1_ITGA6_ITGB41 | 133 | 0.113919 | 1 | receiver |
| IGF2_ITGA6_ITGB41 | 154 | 0.009146 | 1 | receiver |
| CCL5_CCR11 | 125 | 0.227321 | 1 | receiver |
| CCL3_CCR11 | 147 | 0.024124 | 1 | receiver |
| CCL3L1_CCR1 | 98.5 | 1 | 1 | receiver |
| CCL5_CCR31 | 125.5 | 0.214695 | 1 | receiver |
| CCL11_CCR31 | 124.5 | 0.232164 | 1 | receiver |
| CCL28_CCR3 | 98.5 | 1 | 1 | receiver |
| CCL4_CCR51 | 145 | 0.031025 | 1 | receiver |
| CCL5_CCR51 | 125 | 0.227321 | 1 | receiver |
| CCL3_CCR51 | 147 | 0.024124 | 1 | receiver |
| CXCL1_CXCR2 | 98.5 | 1 | 1 | receiver |
| CXCL2_CXCR21 | 99 | 0.969895 | 1 | receiver |
| CXCL3_CXCR21 | 100 | 0.909855 | 1 | receiver |
| CXCL8_CXCR2 | 99 | 0.969895 | 1 | receiver |
| CXCL12_CXCR41 | 134.5 | 0.098058 | 1 | receiver |
| MIF_CD74_CXCR41 | 134 | 0.103523 | 1 | receiver |
| MIF_CD74_CD441 | 136 | 0.084928 | 1 | receiver |
| MIF_CD74_CXCR21 | 131 | 0.137068 | 1 | receiver |
| IL3_IL3RA_CSF2RB1 | 135 | 0.093868 | 1 | receiver |
| CSF2_CSF2RA_CSF2RB1 | 135.5 | 0.089075 | 1 | receiver |
| IL4_IL4R1 | 124 | 0.24563 | 1 | receiver |
| IL4_IL4R_IL13RA11 | 121 | 0.306426 | 1 | receiver |
| IL4_IL4R_IL13RA21 | 124 | 0.24563 | 1 | receiver |
| IL13_IL4R_IL13RA11 | 123 | 0.26491 | 1 | receiver |
| IL13_IL4R_IL13RA21 | 124 | 0.24563 | 1 | receiver |
| IL13_IL13RA11 | 122 | 0.285176 | 1 | receiver |
| IL13_IL13RA21 | 123 | 0.26491 | 1 | receiver |
| IL6_IL6R_IL6ST1 | 147 | 0.024124 | 1 | receiver |
| CNTF_CNTFR_LIFR1 | 155 | 0.007866 | 1 | receiver |
| OSM_LIFR_IL6ST1 | 151 | 0.014108 | 1 | receiver |
| IL1A_IL1R1_IL1RAP1 | 53 | 0.039482 | 1 | receiver |
| IL1B_IL1R1_IL1RAP1 | 61 | 0.093868 | 1 | receiver |
| IL34_CSF1R1 | 149 | 0.018554 | 1 | receiver |
| CSF1_CSF1R1 | 136.5 | 0.080767 | 1 | receiver |
| IL16_CD4 | 98.5 | 1 | 1 | receiver |
| PRL_PRLR1 | 163.5 | 0.002817 | 1 | receiver |
| TNF_TNFRSF1A | 100 | 0.909855 | 1 | receiver |
| TNF_TNFRSF1B | 99 | 0.969895 | 1 | receiver |
| LTA_TNFRSF1B1 | 149 | 0.018554 | 1 | receiver |
| LTA_TNFRSF141 | 149 | 0.018554 | 1 | receiver |
| LTA_LTB_LTBR1 | 142 | 0.044369 | 1 | receiver |
| TNFSF14_LTBR1 | 161 | 0.002963 | 1 | receiver |
| FASL_FAS1 | 150 | 0.016203 | 1 | receiver |
| TNFSF10_TNFRSF10B1 | 135 | 0.093868 | 1 | receiver |
| KLK3_NGFR1 | 156 | 0.006743 | 1 | receiver |
| TNFSF13B_TNFRSF171 | 126.5 | 0.198195 | 1 | receiver |
| TNFSF13B_TNFRSF13B1 | 126.5 | 0.198195 | 1 | receiver |
| TNFSF13B_TNFRSF13C1 | 126.5 | 0.198195 | 1 | receiver |
| CD40LG_ITGA5_ITGB11 | 154 | 0.009146 | 1 | receiver |
| CD40LG_ITGAM_ITGB21 | 149 | 0.018554 | 1 | receiver |
| SPP1_CD44 | 98.5 | 1 | 1 | receiver |
| SPP1_ITGAV_ITGB1 | 98.5 | 1 | 1 | receiver |
| SPP1_ITGAV_ITGB5 | 98.5 | 1 | 1 | receiver |
| SPP1_ITGA4_ITGB1 | 98.5 | 1 | 1 | receiver |
| SPP1_ITGA8_ITGB1 | 98.5 | 1 | 1 | receiver |
| SPP1_ITGA5_ITGB1 | 98.5 | 1 | 1 | receiver |
| NAMPT_INSR1 | 110 | 0.582061 | 1 | receiver |
| NAMPT_ITGA5_ITGB11 | 123 | 0.24098 | 1 | receiver |
| ANGPTL1_ITGA1_ITGB1 | 98.5 | 1 | 1 | receiver |
| ANGPTL2_ITGA5_ITGB1 | 100 | 0.909855 | 1 | receiver |
| ANGPTL2_TLR4 | 99 | 0.969895 | 1 | receiver |
| ANGPT2_ITGA5_ITGB1 | 100 | 0.909855 | 1 | receiver |
| MDK_SDC11 | 104.5 | 0.74775 | 1 | receiver |
| MDK_SDC41 | 105.5 | 0.707505 | 1 | receiver |
| MDK_ITGA4_ITGB11 | 105.5 | 0.707505 | 1 | receiver |
| MDK_ITGA6_ITGB11 | 104.5 | 0.74775 | 1 | receiver |
| MDK_LRP11 | 105.5 | 0.707505 | 1 | receiver |
| MDK_NCL1 | 104.5 | 0.74775 | 1 | receiver |
| MDK_ALK1 | 104.5 | 0.74775 | 1 | receiver |
| PTN_SDC1 | 98.5 | 1 | 1 | receiver |
| PTN_SDC4 | 98.5 | 1 | 1 | receiver |
| PTN_NCL | 98.5 | 1 | 1 | receiver |
| PTN_ALK | 98.5 | 1 | 1 | receiver |
| POSTN_ITGAV_ITGB5 | 100 | 0.909855 | 1 | receiver |
| AGT_AGTR1B | 98.5 | 1 | 1 | receiver |
| C3_CR21 | 152.5 | 0.013083 | 1 | receiver |
| C3_ITGAM_ITGB21 | 158 | 0.004906 | 1 | receiver |
| C3_ITGAX_ITGB21 | 161 | 0.002963 | 1 | receiver |
| C4A_CR21 | 138 | 0.069048 | 1 | receiver |
| PRSS3_F2R1 | 129 | 0.163552 | 1 | receiver |
| CTSG_F2R | 98.5 | 1 | 1 | receiver |
| F2_F2R1 | 153 | 0.0106 | 1 | receiver |
| GZMA_F2R | 101.5 | 0.847727 | 1 | receiver |
| CTSG_F2RL1 | 105 | 0.353111 | 1 | receiver |
| GZMA_F2RL1 | 119 | 0.079464 | 1 | receiver |
| PRSS3_PARD31 | 122 | 0.285176 | 1 | receiver |
| CTSG_PARD3 | 98.5 | 1 | 1 | receiver |
| F2_PARD31 | 142 | 0.044369 | 1 | receiver |
| GZMA_PARD3 | 100.5 | 0.898139 | 1 | receiver |
| NPPC_NPR21 | 147.5 | 0.024338 | 1 | receiver |
| KITL_KIT1 | 139.5 | 0.05955 | 1 | receiver |
| BDNF_NTRK21 | 161 | 0.002963 | 1 | receiver |
| NGF_NTRK11 | 157 | 0.005761 | 1 | receiver |
| FLT3L_FLT31 | 163.5 | 0.002817 | 1 | receiver |
| KLK3_NTRK11 | 152 | 0.012248 | 1 | receiver |
| SEMA3B_NRP1_PLXNA21 | 102.5 | 0.797931 | 1 | receiver |
| SEMA3B_NRP1_PLXNA31 | 101.5 | 0.847727 | 1 | receiver |
| SEMA3C_NRP1_PLXNA21 | 100 | 0.909855 | 1 | receiver |
| SEMA3C_NRP1_PLXNA31 | 100 | 0.909855 | 1 | receiver |
| SEMA3B_NRP2_PLXNA21 | 100.5 | 0.898139 | 1 | receiver |
| SEMA3B_NRP2_PLXNA31 | 100.5 | 0.898139 | 1 | receiver |
| SEMA3C_NRP2_PLXNA21 | 100 | 0.909855 | 1 | receiver |
| SEMA3C_NRP2_PLXNA31 | 100 | 0.909855 | 1 | receiver |
| SEMA3F_NRP2_PLXNA2 | 98.5 | 1 | 1 | receiver |
| SEMA3F_NRP2_PLXNA3 | 98.5 | 1 | 1 | receiver |
| GAS6_AXL1 | 158 | 0.004906 | 1 | receiver |
| GRN_SORT11 | 145 | 0.030652 | 1 | receiver |
| GUCA2A_GUCY2C | 98.5 | 1 | 1 | receiver |
| GUCA2B_GUCY2C | 98.5 | 1 | 1 | receiver |
| PROS1_AXL | 102.5 | 0.797931 | 1 | receiver |
| PSAP_GPR371 | 140 | 0.056205 | 1 | receiver |
| LGALS9_CD441 | 112 | 0.491729 | 1 | receiver |
| COL1A1_ITGA1_ITGB11 | 139 | 0.062042 | 1 | receiver |
| COL1A2_ITGA1_ITGB1 | 100 | 0.909855 | 1 | receiver |
| COL4A1_ITGA1_ITGB1 | 102.5 | 0.797931 | 1 | receiver |
| COL4A2_ITGA1_ITGB1 | 102.5 | 0.797931 | 1 | receiver |
| COL6A1_ITGA1_ITGB1 | 101.5 | 0.847727 | 1 | receiver |
| COL6A2_ITGA1_ITGB1 | 101.5 | 0.847727 | 1 | receiver |
| COL6A3_ITGA1_ITGB1 | 99 | 0.969895 | 1 | receiver |
| COL1A1_ITGA2_ITGB11 | 141 | 0.049736 | 1 | receiver |
| COL1A2_ITGA2_ITGB1 | 100 | 0.909855 | 1 | receiver |
| COL4A1_ITGA2_ITGB1 | 102.5 | 0.797931 | 1 | receiver |
| COL4A2_ITGA2_ITGB1 | 102.5 | 0.797931 | 1 | receiver |
| COL6A1_ITGA2_ITGB1 | 101.5 | 0.847727 | 1 | receiver |
| COL6A2_ITGA2_ITGB1 | 102.5 | 0.797931 | 1 | receiver |
| COL6A3_ITGA2_ITGB1 | 99 | 0.969895 | 1 | receiver |
| FN1_ITGA4_ITGB11 | 141 | 0.049736 | 1 | receiver |
| FN1_ITGA5_ITGB11 | 144 | 0.035045 | 1 | receiver |
| FN1_ITGA8_ITGB11 | 141 | 0.049736 | 1 | receiver |
| LAMA2_ITGA1_ITGB1 | 98.5 | 1 | 1 | receiver |
| LAMA3_ITGA1_ITGB11 | 100 | 0.909855 | 1 | receiver |
| LAMA4_ITGA1_ITGB1 | 102.5 | 0.797931 | 1 | receiver |
| LAMA5_ITGA1_ITGB1 | 100 | 0.909855 | 1 | receiver |
| LAMB1_ITGA1_ITGB11 | 105 | 0.708038 | 1 | receiver |
| LAMB2_ITGA1_ITGB1 | 102.5 | 0.797931 | 1 | receiver |
| LAMB3_ITGA1_ITGB11 | 102.5 | 0.797931 | 1 | receiver |
| LAMC1_ITGA1_ITGB1 | 102.5 | 0.797931 | 1 | receiver |
| LAMC2_ITGA1_ITGB11 | 98.5 | 1 | 1 | receiver |
| LAMA2_ITGA2_ITGB1 | 98.5 | 1 | 1 | receiver |
| LAMA3_ITGA2_ITGB11 | 100 | 0.909855 | 1 | receiver |
| LAMA4_ITGA2_ITGB1 | 102.5 | 0.797931 | 1 | receiver |
| LAMA5_ITGA2_ITGB1 | 100 | 0.909855 | 1 | receiver |
| LAMB1_ITGA2_ITGB11 | 105 | 0.708038 | 1 | receiver |
| LAMB2_ITGA2_ITGB1 | 102.5 | 0.797931 | 1 | receiver |
| LAMB3_ITGA2_ITGB11 | 102.5 | 0.797931 | 1 | receiver |
| LAMC1_ITGA2_ITGB1 | 102.5 | 0.797931 | 1 | receiver |
| LAMC2_ITGA2_ITGB11 | 98.5 | 1 | 1 | receiver |
| LAMA2_ITGA6_ITGB1 | 98.5 | 1 | 1 | receiver |
| LAMA3_ITGA6_ITGB11 | 100 | 0.909855 | 1 | receiver |
| LAMA4_ITGA6_ITGB1 | 102.5 | 0.797931 | 1 | receiver |
| LAMA5_ITGA6_ITGB1 | 100 | 0.909855 | 1 | receiver |
| LAMB1_ITGA6_ITGB11 | 105 | 0.708038 | 1 | receiver |
| LAMB2_ITGA6_ITGB1 | 102.5 | 0.797931 | 1 | receiver |
| LAMB3_ITGA6_ITGB11 | 102.5 | 0.797931 | 1 | receiver |
| LAMC1_ITGA6_ITGB1 | 102.5 | 0.797931 | 1 | receiver |
| LAMC2_ITGA6_ITGB11 | 98.5 | 1 | 1 | receiver |
| LAMA2_ITGA7_ITGB1 | 98.5 | 1 | 1 | receiver |
| LAMA3_ITGA7_ITGB11 | 100 | 0.909855 | 1 | receiver |
| LAMA4_ITGA7_ITGB1 | 102.5 | 0.797931 | 1 | receiver |
| LAMA5_ITGA7_ITGB1 | 100 | 0.909855 | 1 | receiver |
| LAMB1_ITGA7_ITGB11 | 105 | 0.708038 | 1 | receiver |
| LAMB2_ITGA7_ITGB1 | 102.5 | 0.797931 | 1 | receiver |
| LAMB3_ITGA7_ITGB11 | 102.5 | 0.797931 | 1 | receiver |
| LAMC1_ITGA7_ITGB1 | 102.5 | 0.797931 | 1 | receiver |
| LAMC2_ITGA7_ITGB11 | 98.5 | 1 | 1 | receiver |
| TNC_ITGA8_ITGB1 | 100 | 0.909855 | 1 | receiver |
| FN1_ITGAV_ITGB11 | 158 | 0.004906 | 1 | receiver |
| FN1_ITGA4_ITGB71 | 142 | 0.044369 | 1 | receiver |
| LAMA2_ITGA6_ITGB4 | 98.5 | 1 | 1 | receiver |
| LAMA3_ITGA6_ITGB41 | 100 | 0.909855 | 1 | receiver |
| LAMA4_ITGA6_ITGB4 | 102.5 | 0.797931 | 1 | receiver |
| LAMA5_ITGA6_ITGB4 | 100 | 0.909855 | 1 | receiver |
| LAMB1_ITGA6_ITGB41 | 104 | 0.751336 | 1 | receiver |
| LAMB2_ITGA6_ITGB4 | 102.5 | 0.797931 | 1 | receiver |
| LAMB3_ITGA6_ITGB41 | 102.5 | 0.797931 | 1 | receiver |
| LAMC1_ITGA6_ITGB4 | 102.5 | 0.797931 | 1 | receiver |
| LAMC2_ITGA6_ITGB41 | 98.5 | 1 | 1 | receiver |
| FN1_CD441 | 145 | 0.031025 | 1 | receiver |
| COL1A1_CD441 | 142 | 0.044369 | 1 | receiver |
| COL1A2_CD44 | 100 | 0.909855 | 1 | receiver |
| COL4A1_CD44 | 102.5 | 0.797931 | 1 | receiver |
| COL4A2_CD44 | 102.5 | 0.797931 | 1 | receiver |
| COL6A1_CD44 | 101.5 | 0.847727 | 1 | receiver |
| COL6A2_CD44 | 102.5 | 0.797931 | 1 | receiver |
| COL6A3_CD44 | 99 | 0.969895 | 1 | receiver |
| LAMA2_CD44 | 98.5 | 1 | 1 | receiver |
| LAMA3_CD441 | 100 | 0.909855 | 1 | receiver |
| LAMA4_CD44 | 102.5 | 0.797931 | 1 | receiver |
| LAMA5_CD44 | 100 | 0.909855 | 1 | receiver |
| LAMB1_CD441 | 106 | 0.665664 | 1 | receiver |
| LAMB2_CD44 | 102.5 | 0.797931 | 1 | receiver |
| LAMB3_CD441 | 102.5 | 0.797931 | 1 | receiver |
| LAMC1_CD44 | 102.5 | 0.797931 | 1 | receiver |
| LAMC2_CD441 | 98.5 | 1 | 1 | receiver |
| COL1A1_SDC11 | 133 | 0.113919 | 1 | receiver |
| COL1A2_SDC1 | 100 | 0.909855 | 1 | receiver |
| COL4A1_SDC1 | 102.5 | 0.797931 | 1 | receiver |
| COL4A2_SDC1 | 102.5 | 0.797931 | 1 | receiver |
| COL6A1_SDC1 | 100.5 | 0.898139 | 1 | receiver |
| COL6A2_SDC1 | 101.5 | 0.847727 | 1 | receiver |
| COL6A3_SDC1 | 99 | 0.969895 | 1 | receiver |
| FN1_SDC11 | 135 | 0.093868 | 1 | receiver |
| TNC_SDC1 | 100 | 0.909855 | 1 | receiver |
| THBS1_SDC11 | 131 | 0.137068 | 1 | receiver |
| THBS2_SDC1 | 98.5 | 1 | 1 | receiver |
| COL1A1_SDC41 | 141 | 0.049736 | 1 | receiver |
| COL1A2_SDC4 | 100 | 0.909855 | 1 | receiver |
| COL4A1_SDC4 | 102.5 | 0.797931 | 1 | receiver |
| COL4A2_SDC4 | 102.5 | 0.797931 | 1 | receiver |
| COL6A1_SDC4 | 101.5 | 0.847727 | 1 | receiver |
| COL6A2_SDC4 | 102.5 | 0.797931 | 1 | receiver |
| COL6A3_SDC4 | 99 | 0.969895 | 1 | receiver |
| FN1_SDC41 | 144 | 0.035045 | 1 | receiver |
| TNC_SDC4 | 100 | 0.909855 | 1 | receiver |
| THBS1_SDC41 | 138 | 0.069048 | 1 | receiver |
| THBS2_SDC4 | 98.5 | 1 | 1 | receiver |
| THBS1_CD471 | 139 | 0.062042 | 1 | receiver |
| THBS2_CD47 | 98.5 | 1 | 1 | receiver |
| AGRN_DAG11 | 112 | 0.164915 | 1 | receiver |
| LAMA2_DAG1 | 105 | 0.353111 | 1 | receiver |
| LAMA3_DAG11 | 112 | 0.164915 | 1 | receiver |
| LAMA4_DAG1 | 119 | 0.079464 | 1 | receiver |
| LAMA5_DAG1 | 112 | 0.164915 | 1 | receiver |
| LAMB1_DAG11 | 126 | 0.037919 | 1 | receiver |
| LAMB2_DAG1 | 119 | 0.079464 | 1 | receiver |
| LAMB3_DAG11 | 119 | 0.079464 | 1 | receiver |
| LAMC1_DAG1 | 119 | 0.079464 | 1 | receiver |
| LAMC2_DAG11 | 105 | 0.353111 | 1 | receiver |
| ALCAM_CD61 | 147 | 0.003392 | 1 | receiver |
| APP_CD741 | 118 | 0.376095 | 1 | receiver |
| CD40LG_CD401 | 142.5 | 0.043179 | 1 | receiver |
| PTPRC_CD22 | 121 | 0.281559 | 1 | receiver |
| CD48_CD244A | 113 | 0.487712 | 1 | receiver |
| CD6_ALCAM1 | 147 | 0.003392 | 1 | receiver |
| CD80_CD281 | 140 | 0.055617 | 1 | receiver |
| CD80_CTLA41 | 136 | 0.084928 | 1 | receiver |
| CD86_CD281 | 146 | 0.027396 | 1 | receiver |
| CD86_CTLA41 | 140 | 0.055617 | 1 | receiver |
| CDH2_CDH21 | 140.5 | 0.053599 | 1 | receiver |
| CDH1_CDH11 | 147.5 | 0.024338 | 1 | receiver |
| CDH1_ITGA1_ITGB11 | 152 | 0.012248 | 1 | receiver |
| CDH1_KLRG11 | 146 | 0.027396 | 1 | receiver |
| CDH5_CDH51 | 139.5 | 0.05955 | 1 | receiver |
| CEACAM1_CEACAM51 | 112 | 0.164915 | 1 | receiver |
| DSC2_DSG21 | 105 | 0.708038 | 1 | receiver |
| EFNA1_EPHA11 | 138 | 0.069048 | 1 | receiver |
| EFNA1_EPHA21 | 141 | 0.049736 | 1 | receiver |
| EFNA1_EPHA31 | 126 | 0.209978 | 1 | receiver |
| EFNA1_EPHA41 | 126 | 0.209978 | 1 | receiver |
| EFNA1_EPHA71 | 134 | 0.103523 | 1 | receiver |
| EFNA2_EPHA11 | 139 | 0.062042 | 1 | receiver |
| EFNA2_EPHA21 | 142 | 0.044369 | 1 | receiver |
| EFNA2_EPHA31 | 128 | 0.178112 | 1 | receiver |
| EFNA2_EPHA41 | 128 | 0.178112 | 1 | receiver |
| EFNA2_EPHA71 | 135 | 0.093868 | 1 | receiver |
| EFNA4_EPHA11 | 143 | 0.039482 | 1 | receiver |
| EFNA4_EPHA21 | 146 | 0.027396 | 1 | receiver |
| EFNA4_EPHA31 | 131 | 0.137068 | 1 | receiver |
| EFNA4_EPHA41 | 131 | 0.137068 | 1 | receiver |
| EFNA4_EPHA71 | 138 | 0.069048 | 1 | receiver |
| EFNA5_EPHA11 | 138 | 0.069048 | 1 | receiver |
| EFNA5_EPHA21 | 141 | 0.049736 | 1 | receiver |
| EFNA5_EPHA31 | 129.5 | 0.154281 | 1 | receiver |
| EFNA5_EPHA41 | 131 | 0.137068 | 1 | receiver |
| EFNA5_EPHA71 | 136 | 0.084928 | 1 | receiver |
| EFNA5_EPHB21 | 154 | 0.009146 | 1 | receiver |
| EFNB1_EPHA41 | 100.5 | 0.898139 | 1 | receiver |
| EFNB1_EPHB21 | 102.5 | 0.797931 | 1 | receiver |
| EFNB1_EPHB31 | 119 | 0.079464 | 1 | receiver |
| EFNB1_EPHB41 | 101.5 | 0.847727 | 1 | receiver |
| EFNB2_EPHA41 | 128 | 0.178112 | 1 | receiver |
| EFNB2_EPHB21 | 150 | 0.016203 | 1 | receiver |
| EFNB2_EPHB41 | 132 | 0.125092 | 1 | receiver |
| EFNB3_EPHA41 | 127 | 0.193579 | 1 | receiver |
| EFNB3_EPHB21 | 149 | 0.018554 | 1 | receiver |
| EFNB3_EPHB41 | 133 | 0.113919 | 1 | receiver |
| ICAM1_ITGAX_ITGB21 | 155 | 0.007866 | 1 | receiver |
| ICAM1_ITGAL_ITGB21 | 155 | 0.007866 | 1 | receiver |
| ICAM2_ITGAL_ITGB2 | 100 | 0.909855 | 1 | receiver |
| ICAM1_ITGAM_ITGB21 | 151 | 0.014108 | 1 | receiver |
| ICAM2_ITGAM_ITGB2 | 100 | 0.909855 | 1 | receiver |
| ICAM1_ITGAL1 | 154 | 0.009146 | 1 | receiver |
| ITGB2_ICAM11 | 159 | 0.004162 | 1 | receiver |
| JAM2_ITGAV_ITGB1 | 98.5 | 1 | 1 | receiver |
| JAM3_ITGAM_ITGB2 | 102.5 | 0.797931 | 1 | receiver |
| JAM1_ITGAL_ITGB21 | 108.5 | 0.591949 | 1 | receiver |
| F11R_F11R1 | 106 | 0.687621 | 1 | receiver |
| JAM2_F11R | 98.5 | 1 | 1 | receiver |
| JAM3_F11R | 102.5 | 0.797931 | 1 | receiver |
| HLA-A_CD8A1 | 152 | 0.012248 | 1 | receiver |
| HLA-B_CD8A1 | 148 | 0.021187 | 1 | receiver |
| HLA-C_CD8A1 | 150 | 0.016203 | 1 | receiver |
| HLA-E_CD8A1 | 150 | 0.016203 | 1 | receiver |
| HLA-F_CD8A1 | 141 | 0.05053 | 1 | receiver |
| HLA-C_KIR2DL31 | 149.5 | 0.019096 | 1 | receiver |
| HLA-DPA1_CD4 | 112 | 0.518219 | 1 | receiver |
| HLA-DPB1_CD4 | 107 | 0.665081 | 1 | receiver |
| HLA-DQA1_CD4 | 103 | 0.795432 | 1 | receiver |
| HLA-DMA_CD4 | 101 | 0.885472 | 1 | receiver |
| HLA-DMB_CD4 | 100.5 | 0.898139 | 1 | receiver |
| HLA-DQA2_CD4 | 102.5 | 0.797931 | 1 | receiver |
| HLA-DOA_CD4 | 102.5 | 0.797931 | 1 | receiver |
| HLA-DOB_CD4 | 102.5 | 0.797931 | 1 | receiver |
| HLA-DQB1_CD4 | 103 | 0.795432 | 1 | receiver |
| HLA-DRA_CD4 | 106 | 0.702477 | 1 | receiver |
| HLA-DRB1_CD41 | 125.5 | 0.214695 | 1 | receiver |
| HLA-DRB5_CD4 | 103.5 | 0.788694 | 1 | receiver |
| MPZL1_MPZL11 | 136.5 | 0.080767 | 1 | receiver |
| DLL1_NOTCH11 | 100 | 0.909855 | 1 | receiver |
| DLL1_NOTCH31 | 100 | 0.909855 | 1 | receiver |
| DLL4_NOTCH1 | 98.5 | 1 | 1 | receiver |
| DLL4_NOTCH3 | 98.5 | 1 | 1 | receiver |
| JAG2_NOTCH1 | 98.5 | 1 | 1 | receiver |
| JAG2_NOTCH3 | 98.5 | 1 | 1 | receiver |
| SELL_PODXL | 99 | 0.969895 | 1 | receiver |
| SEMA4A_NRP1_PLXNA2 | 98.5 | 1 | 1 | receiver |
| SEMA4A_NRP1_PLXNA3 | 98.5 | 1 | 1 | receiver |
| SEMA4A_PLXNB1 | 98.5 | 1 | 1 | receiver |
| SEMA4A_PLXNB2 | 98.5 | 1 | 1 | receiver |
| SEMA4D_PLXNB21 | 144 | 0.035045 | 1 | receiver |
| SEMA4C_PLXNB2 | 100 | 0.909855 | 1 | receiver |
| SEMA5A_PLXNA31 | 100.5 | 0.898139 | 1 | receiver |
| SEMA6A_PLXNA2 | 99 | 0.969895 | 1 | receiver |
| SEMA6B_PLXNA2 | 100 | 0.909855 | 1 | receiver |
| SEMA7A_ITGB1_ITGA1 | 101.5 | 0.847727 | 1 | receiver |
| THY1_ITGAM_ITGB21 | 139 | 0.062042 | 1 | receiver |
| THY1_ITGAX_ITGB21 | 144 | 0.035045 | 1 | receiver |
| VSIR_IGSF11 | 108 | 0.6285 | 1 | receiver |

**Supplementary table 3**

The 19 candidate genes of KRD score.

| symbol | coef |
| --- | --- |
| KTI12 | -0.58315 |
| CCL22 | -0.34627 |
| RNFT1 | -0.11594 |
| KLK12 | -0.09509 |
| OIT3 | -0.06192 |
| SPINK4 | -0.03742 |
| MFHAS1 | -0.02481 |
| MMP3 | -0.01612 |
| CBX7 | 0.008904 |
| MYOF | 0.035671 |
| HOXC11 | 0.058233 |
| NPDC1 | 0.072458 |
| NPIPB5 | 0.084393 |
| SMARCD3 | 0.099179 |
| RAB3IL1 | 0.099349 |
| WIPF3 | 0.114216 |
| JDP2 | 0.161337 |
| PRRT2 | 0.230167 |
| CCDC32 | 0.428901 |
